# Molecular and Cellular Underpinnings of Primary Hypertension: An Integrative Mechanistic Framework

**DOI:** 10.1002/jcp.70233

**Published:** 2026-09-29

**Authors:** Dima Nasrallah, Alaa Abdelhamid, Omar Tluli, Yaman Al‐Haneedi, Mera Abu Hussien, Ali H. Eid

**Affiliations:** ^1^ College of Medicine, QU Health Qatar University Doha Qatar; ^2^ School of Medicine The University of Jordan Amman Jordan; ^3^ Department of Basic Medical Sciences, College of Medicine, QU Health Qatar University Doha Qatar

**Keywords:** diet, hypertension, inflammation, insulin resistance, microbiota dysbiosis, oxidative stress, RAAS

## Abstract

Hypertension is a highly prevalent clinical condition and a major contributor to global cardiovascular morbidity and mortality. Despite extensive research, its complex and multifaceted pathogenesis is yet to be fully elucidated. This paper explores the updated understanding of primary hypertension as a systemic disorder rather than an isolated vascular or renal abnormality. At the center of this multifactorial disease lie three primary and interrelated pathophysiological mechanisms: dysregulation of the renin–angiotensin–aldosterone system, dysfunction of the central nervous system, and activation of inflammatory and oxidative stress pathways. These core drivers not only interact with each other in a self‐reinforcing cycle but also serve as convergence points for a range of additional contributors. Specifically, suboptimal dietary patterns, salt sensitivity, gut microbiota dysbiosis, circadian rhythm disruption, and insulin resistance act by modulating these central processes, thereby perpetuating blood pressure elevation. This review further elaborates on how these mechanisms span across multiple organ systems, driving vascular, renal, neurohumoral, and immune dysfunction. Taken together, this perspective underscores the need for integrated, upstream‐targeted therapeutic strategies. By highlighting the interconnected nature of these drivers and contributors, this paper provides a comprehensive framework to inform future research and guide more effective preventive and management approaches for hypertension.

**Abbreviations**
ACC: American College of Cardiology
ACE: angiotensin‐converting enzyme
ADD1: α‐adducin 1
ADH: antidiuretic hormone
AGEs: advanced glycation end‐products
AP‐1: activator protein‐1
ARBs: angiotensin‐II receptor blockers
AT_1_R: angiotensin‐II receptor type 1
BMAL1: brain and muscle ARNT‐like 1
cAMP: cyclic adenosine monophosphate
CCGs: clock‐controlled genes
CCL2: chemokine (C‐C motif) ligand 2
CLOCK: circadian locomotor output cycles protein kaput
CNS: central nervous system
CRP: C‐reactive protein
CRY: cryptochrome
CVDs: cardiovascular diseases
CVLM: caudal ventrolateral medulla
DASH: Dietary Approaches to Stop Hypertension
DBP: diastolic blood pressure
DCs: dendritic cells
ENaC: epithelial sodium channel
eNOS: endothelial nitric oxide synthase
ESC: European Society of Cardiology
ESH: European Society of Hypertension
ET‐1: endothelin‐1
FFA: free fatty acid
FMT: fecal microbiota transfer
F:B: Firmicutes‐to‐Bacteroidetes
GABA: gamma‐aminobutyric acid
GPR41: G‐protein‐coupled receptor 41
GPR43: G‐protein‐coupled receptor 43
GRK4: G protein–coupled receptor kinase 4
HMGB1: high‐mobility group box 1
ICAM‐1: intercellular adhesion molecule‐1
IL: interleukin
IR: insulin receptor
IRS: insulin receptor substrate
isoLGs: isolevuglandins
LPS: lipopolysaccharide
LVH: left ventricular hypertrophy
MAP: mean arterial pressure
MAPK: mitogen‐activated protein kinase
MLCP: myosin light chain phosphatase
MyD88: myeloid differentiation primary response 88
NADPH: nicotinamide adenine dinucleotide phosphate
NCC: Na^+^‐Cl^−^ cotransporter
NF‐κB: nuclear factor kappa B
NKCC2: Na^+^‐K^+^−2Cl^−^ cotransporter
NO: nitric oxide
NOX: NADPH oxidase
NSAIDs: nonsteroidal anti‐inflammatory drugs
NTS: nucleus tractus solitarius
NT‐proBNP: N‐terminal pro B‐type natriuretic peptide
PER: period
PI3K: phosphoinositide 3‐kinase
PVN: paraventricular nucleus
RAAS: renin–angiotensin–aldosterone system
RAGE: receptor for advanced glycation end‐products
RHT: retinohypothalamic tract
ROS: reactive oxygen species
RVLM: rostral ventrolateral medulla
SBP: systolic blood pressure
SCFA: short‐chain fatty acid
SCN: suprachiasmatic nucleus
SNA: sympathetic nerve activity
SOX6: SRY‐box transcription factor 6
TGF‐β: transforming growth factor‐β
Th‐1: T helper 1
Th‐17: T helper 17
TLR4: toll‐like receptor 4
TNF‐α: tumor necrosis factor alpha
TTFL: transcription–translation feedback loop
VCAM‐1: vascular cell adhesion molecule‐1
VSMCs: vascular smooth muscle cells
WHO: World Health Organization

## 1 Introduction

Cardiovascular disease (CVD) represents an escalating global public health burden, accounting for a disproportionately high share of global morbidity and mortality (Roth et al. 2020). According to the World Health Organization (WHO), ischemic heart disease has remained the leading cause of death worldwide for over three decades (World Health Organization 2024). Among the key drivers of this burden is hypertension, a major cardiovascular condition and an important modifiable risk factor for ischemic heart disease amongst various other cardiovascular disorders. In 2023, the global prevalence of hypertension was estimated at approximately 1.28 billion adults, with projections indicating a further rise to 1.56 billion by 2025, underscoring its urgent public health implications (Chockalingam 2007; World Health Organization 2023). Alarmingly, nearly half of affected individuals remain undiagnosed, and for those diagnosed, treatment often fails to achieve adequate control (Mills et al. 2016). This widespread gap in detection and effective management substantially amplifies the risk of adverse cardiovascular sequalae, including heart failure, stroke, and ultimately premature death (Mills et al. 2016).

Hypertension is a chronic condition defined by persistently elevated systemic arterial blood pressure, underpinned by a multifaceted and dynamic interplay of pathophysiological mechanisms (Oparil et al. 2018). Notably, there is ongoing divergence among major international societies regarding the diagnostic threshold for hypertension. The 2025 American College of Cardiology and American Heart Association (ACC/AHA) guidelines designates a systolic blood pressure (SBP) ≥ 130 mmHg or diastolic blood pressure (DBP) ≥ 80 mmHg as hypertensive (Jones et al. 2025). In contrast, the 2023/2024 European Society of Cardiology (ESC) and European Society of Hypertension (ESH) guidelines set the threshold at SBP ≥ 140 mmHg or DBP ≥ 90 mmHg. Blood pressure readings falling between 130/80 and 140/90 are classified as “high normal” or “elevated” in the European system (B. X. Wang 2025). Nonetheless, both consensus statements stipulate that a diagnosis be established on the basis of at least two elevated readings obtained on separate occasions, preferably outside the clinic setting, using ambulatory or home blood pressure monitoring rather than office readings (Jones et al. 2025; B. X. Wang 2025).

Hypertension can be broadly classified as either primary (essential) or secondary (Oparil et al. 2018). Primary hypertension arises from complex pathogenesis involving genetic, environmental, and systemic influences, while secondary hypertension denotes an elevated blood pressure attributable to a specific, often reversible underlying cause (Oparil et al. 2018). Some common causes include renal disorders such as renal artery stenosis; endocrine abnormalities like hyperthyroidism; congenital cardiovascular anomalies such as coarctation of the aorta; and iatrogenic factors such as oral contraceptives, nonsteroidal anti‐inflammatory drugs (NSAIDs) and glucocorticoids (Oparil et al. 2018).

The etiological ambiguity of primary hypertension has stimulated decades of research exploring its complex pathophysiology. Initially, investigators tended to focus on singular organ system, vascular, renal, or cardiac, as the principal site of dysfunction. However, this reductionist perspective underappreciates the multifactorial nature of hypertension and fails to capture the full spectrum of its systemic impact. In 1949, Dr. Irvine Page articulated the “mosaic theory of hypertension,” arguing that elevated blood pressure arises from the combined and interactive effects of multiple physiological (Page 1949). Originally, the mosaic theory enumerated eight distinct entities: chemical influences, neural activity, arterial elasticity, cardiac output, blood viscosity, vascular caliber, blood volume, and vascular reactivity (Page 1949). These were later revised to encompass broader categories such as genetic, hemodynamic, humoral, endocrine, neural, adaptive, anatomical, and environmental factors (Harrison et al. 2021). The evolution of this theory underscores the complexity of hypertension and supports a systems‐level approach to understanding its pathogenesis. With evolving scientific insight, the mosaic theory has recently been revisited and expanded to incorporate additional elements such as the immune system and gut microbiota (Harrison et al. 2021). While this updated framework offers crucial integrative perspectives, it falls short of addressing the potential complexity and dynamic nature of the interactions among these factors.

In light of the aforementioned gaps in current models, this review seeks to clarify the central drivers and unifying mechanisms that orchestrate the diverse physiological disturbances underlying primary hypertension. Foremost among these are dysregulation of the renin–angiotensin–aldosterone system (RAAS), neural mechanisms, particularly sympathetic nerve overactivity, and heightened inflammatory and oxidative stress states, which together represent the core pathological axes of the disease. Additional contributing factors such as suboptimal dietary patterns, salt sensitivity, alterations in gut microbiota composition, circadian rhythm disturbances, and insulin resistance, primarily serve to initiate or augment these core pathways. This conceptual framework, which views hypertension as a manifestation of interdependent dysfunction across multiple organ systems, offers a more integrated understanding of its pathogenesis and may inform more targeted approaches to its management.

## 2 Dysregulation of Renin–Angiotensin–Aldosterone System

The homeostatic control of blood pressure, extracellular fluid volume, and serum sodium concentration depends on a sophisticated network of interdependent processes governed principally by the renal and cardiovascular systems. Within this framework, the kidney contributes to blood pressure regulation through four major mechanisms: modulation of sodium and water excretion via pressure–natriuresis, endocrine regulation through renin synthesis and release, integration of neural reflex pathways via renal afferent signaling, and participation in immune and inflammatory activation that influences vascular and renal function. Together, these mechanisms enable the kidney to coordinate long‐term volume balance and systemic hemodynamics, thereby exerting a dominant influence on arterial pressure control (Harrison et al. 2021). Central to this regulatory nexus is the RAAS, a key mediator of vascular and fluid homeostasis (Patel et al. 2017) Renin release represents the initial and rate‐limiting step within the axis (Figure 1), and is primarily triggered by reductions in renal perfusion pressure, decreased distal sodium delivery sensed by the macula densa, and heightened sympathetic stimulation of juxtaglomerular cells via β_1_‐adrenergic receptors (Patel et al. 2017). Upon release, renin cleaves hepatic angiotensinogen into angiotensin I, which is subsequently converted by angiotensin‐converting enzyme (ACE) into angiotensin II, the principal effector peptide of RAAS (Navar 2014). Angiotensin II, binding to angiotensin II receptor type 1 (AT_1_R), orchestrates systemic vasoconstriction, thereby raising total peripheral resistance and arterial pressure (Moon 2013). It preferentially constricts efferent renal arterioles to preserve glomerular filtration, and stimulates aldosterone secretion to enhance sodium and water retention (Navar 2014). In extreme cases of hypotension, angiotensin II can also act on the hypothalamus to trigger thirst and promote antidiuretic hormone (ADH) release from the posterior pituitary, which augments water reabsorption in collecting ducts (Patel et al. 2017). Collectively, these actions function to expand plasma volume and elevate arterial blood pressure.

**Figure 1:**
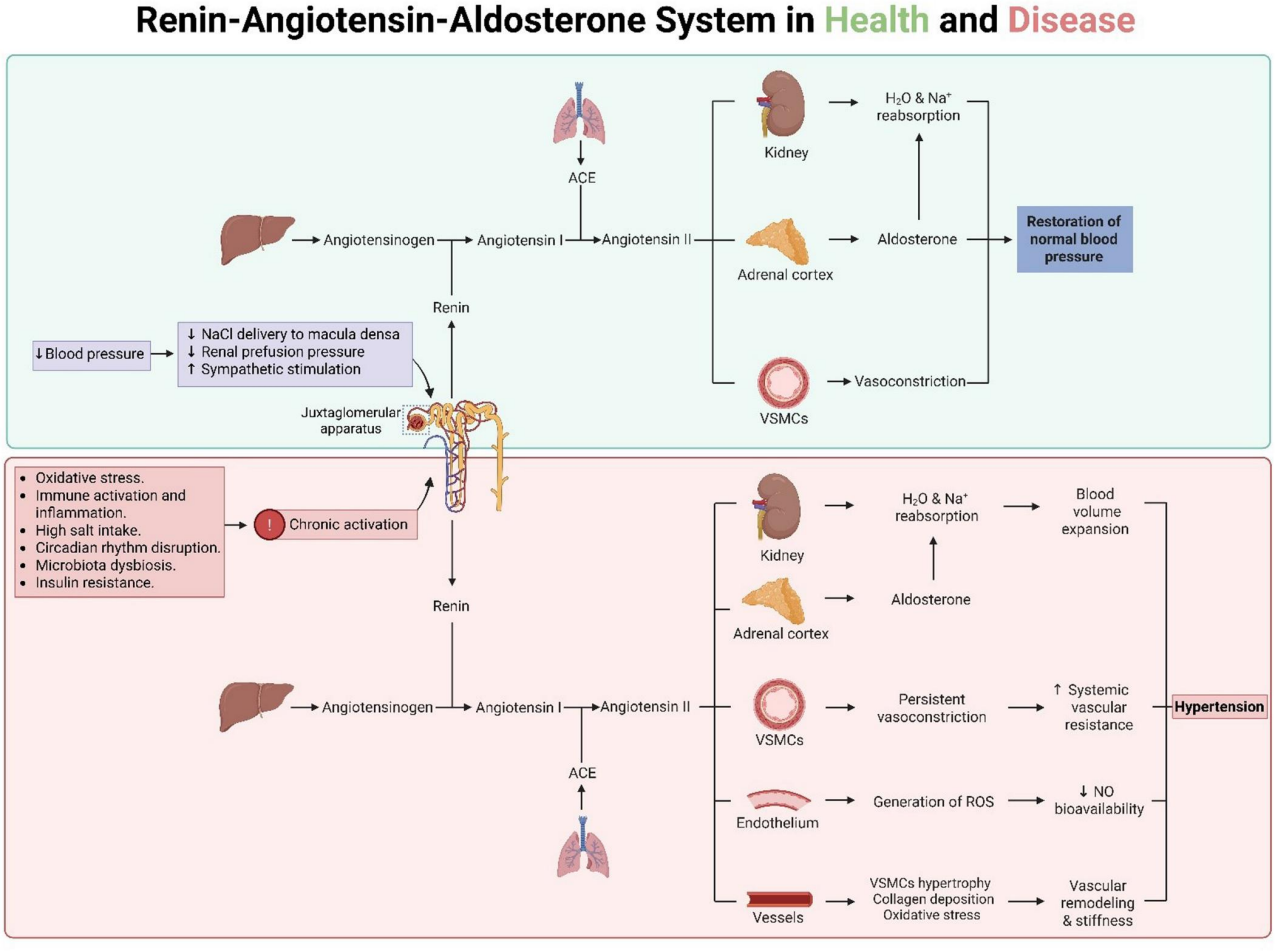
Overview of the renin–angiotensin–aldosterone system (RAAS) under physiological conditions compared to hypertension. In healthy states, RAAS activation is triggered by decreased NaCl delivery to the macula densa, reduced renal perfusion pressure, and increased sympathetic stimulation, responses secondary to a drop in blood pressure. This activates angiotensin II–mediated vasoconstriction and aldosterone‐driven sodium and water reabsorption, ultimately restoring normotension. In disease, chronic RAAS activation, fueled by various factors, including oxidative stress, inflammation, microbiota dysbiosis, circadian disruption, and insulin resistance, causes persistent water and sodium reabsorption, vasoconstriction, endothelial ROS generation, and vascular remodeling. These changes increase systemic vascular resistance and blood volume, ultimately leading to hypertension. Created with BioRender.com.

While RAAS activation is critical for restoring circulatory homeostasis during hypovolemia or hypotension, its chronic stimulation promotes pathological sodium retention, vascular dysfunction, inflammation, and cardiovascular remodeling, which are hallmarks of hypertension (Patel et al. 2017). Experimental models show that sustained RAAS stimulation induces cellular plasticity evident by the transformation of afferent arteriolar smooth muscle cells into renin‐producing cells (Saleem et al. 2020). Moreover, under conditions of oxidative stress, decreased nitric oxide (NO) bioavailability, and sympathetic overactivity, even proximal tubular epithelial cells can adopt a renin‐producing phenotype, thus amplifying local and systemic RAAS expression and promoting the maladaptive changes linked to hypertension (Harrison et al. 2021). Consistent with these findings, clinical evidence from 36 patients with primary aldosteronism further supports the contribution of sustained aldosterone signaling to vascular remodeling (Strauch et al. 2006). Patients with primary aldosteronism exhibited significantly greater arterial stiffness than those with essential hypertension despite comparable office blood pressure (Strauch et al. 2006). These findings suggest that excess aldosterone may exert direct deleterious effects on the arterial wall beyond those attributable to elevated blood pressure alone.

The RAAS is intricately intertwined with the sympathetic nervous system, establishing a bidirectional and self‐amplifying circuit that fundamentally contributes to the pathogenesis of hypertension (Grassi et al. 2005). Indeed, evidence shows that central administration of angiotensin II robustly augments sympathetic outflow, particularly from hypothalamic nuclei, thereby increasing peripheral vascular resistance and accelerating heart rate (Manrique et al. 2009). This central effect is complemented by peripheral actions, where angiotensin II not only facilitates neurotransmission at sympathetic ganglia but also impedes norepinephrine reuptake at adrenergic nerve terminals, thus amplifying adrenergic signaling and perpetuating vasoconstriction (Grassi et al. 2005). The clinical significance of these mechanisms is underscored by the observation that ACE inhibition mitigates this adrenergic potentiation, enhancing norepinephrine clearance and offering beneficial effects beyond hemodynamic modulation (Manrique et al. 2009). Bridging these regulatory pathways, the sympathetic nervous system reciprocally drives RAAS activation through β_1_‐adrenergic receptors‐mediated renin release from juxtaglomerular cells (Patel et al. 2017). By establishing a feed‐forward loop, RAAS and sympathetic system sustain pathologically elevated blood pressure, illustrating a pivotal mechanistic axis in hypertension.

However, the strong interaction between angiotensin II and sympathetic activation observed in experimental models has not been consistently reproduced in humans. It was found that infusion of angiotensin II in healthy volunteers did not increase circulating adrenaline or noradrenaline, and suppressing angiotensin II with captopril in patients with severe hypertension failed to reduce catecholamine levels (Nicholls et al. 1981). Similarly, angiotensin II infusion in six healthy volunteers failed to significantly increase basal sympathetic activity as assessed by plasma noradrenaline and vital signs (Goldsmith and Hasking 1990). These discrepancies may partly reflect the substantially higher angiotensin II doses used in animal experiments, whereas human studies typically utilize physiological or subpressor concentrations that may be insufficient to elicit detectable sympathetic activation (Grassi et al. 2015). Moreover, circulating catecholamines and global noradrenaline spillover may not adequately capture localized sympathetic effects within specific vascular or neural beds, limiting direct translation of the proposed mechanisms to humans (Grassi et al. 2015). Thus, while RAAS–sympathetic interactions are well established experimentally, their magnitude and physiological relevance in human hypertension remain incompletely defined.

Accumulating evidence further delineates the impact of chronic RAAS activation on the development of hypertension through oxidative stress–mediated mechanisms (Manrique et al. 2009). Angiotensin II, acting through AT_1_R, stimulates nicotinamide adenine dinucleotide phosphate (NADPH) oxidase (NOX), leading to excessive production of reactive oxygen species (ROS). These ROS are potent scavengers of NO, converting it into peroxynitrite and thereby reducing NO bioavailability, a critical determinant of vasotone and endothelial health (Manrique et al. 2009). Aldosterone independently amplifies this deleterious process by activating NOX pathways (Manrique et al. 2009). Moreover, RAAS‐induced oxidative stress not only limits the availability of tetrahydrobiopterin (BH_4_), an essential cofactor for NO synthases, but also depletes L‐arginine, the substrate for NO synthesis (Tirapelli 2021). Consequently, endothelial nitric oxide synthase (eNOS) becomes uncoupled, favoring superoxide production over NO and further exacerbating oxidative stress and vascular dysfunction (Tirapelli 2021). The resultant impairment in vasodilatory capacity perpetuates endothelial injury, contributing to sustained elevations in blood pressure (Tirapelli 2021).

Importantly, chronic RAAS overactivation is not merely a downstream consequence of circulatory stress but is often driven by a range of upstream pathological stimuli. Factors such as suboptimal dietary patterns, insulin resistance, circadian rhythm disruptions, and microbiota dysbiosis are all implicated in the persistent stimulation of RAAS, albeit through diverse mechanisms (Figure 1). These contributors, despite their distinct origins, ultimately converge to drive a pathogenic continuum characterized by sodium retention, plasma volume expansion, and progressive vascular remodeling. Collectively, these processes represent the cardinal features of hypertension and highlight the centrality of RAAS in both the initiation and maintenance of hypertensive disease states. Accordingly, inhibition of the RAAS axis remains the cornerstone of antihypertensive pharmacotherapy. Agents such as ACE inhibitors, angiotensin II receptor blockers (ARBs), and mineralocorticoid receptor antagonists attenuate maladaptive RAAS‐signaling and exert robust antihypertensive effects (Patel et al. 2017). Recent therapeutic strategies have also targeted endothelin signaling pathways, such as the dual endothelin receptor antagonist aprocitentan, which has demonstrated efficacy in resistant hypertension by reducing endothelin‐mediated vasoconstriction and vascular remodeling (Sahebkar and Eid 2025).

## 3 Sympathetic Nerve Overactivity

The central nervous system (CNS) plays an important role in maintaining blood pressure homeostasis, integrating complex interactions with other regulatory systems such as the RAAS and immune mechanisms. This is primarily achieved by modulating autonomic outflow in response to afferent signals originating from baroreceptors (Figure 2). These mechanosensitive receptors, located in the aortic arch and carotid sinus, detect changes in arterial wall stretch as surrogates for blood pressure alterations (Patel et al. 2017). An increase in arterial pressure augments vascular wall stretch, leading to elevated baroreceptor firing. These sensory signals are conveyed via the glossopharyngeal (cranial nerve IX) and vagus (cranial nerve X) nerves to the nucleus tractus solitarius (NTS) in the medulla, the principal cardiovascular reflex integration center (Struthers and Dollery 1985). Upon receiving heightened afferent activity, the NTS orchestrates an increase in parasympathetic output through the nucleus ambiguous and dorsal motor nucleus of the vagus. Concurrently, it suppresses sympathetic outflow via inhibitory projections to the caudal and rostral ventrolateral (CVLM and RVLM) medulla. This coordinated autonomic modulation results in decreased heart rate, reduced stroke volume, and peripheral vasodilation, collectively restoring arterial pressure to its homeostatic range (Struthers and Dollery 1985).

**Figure 2:**
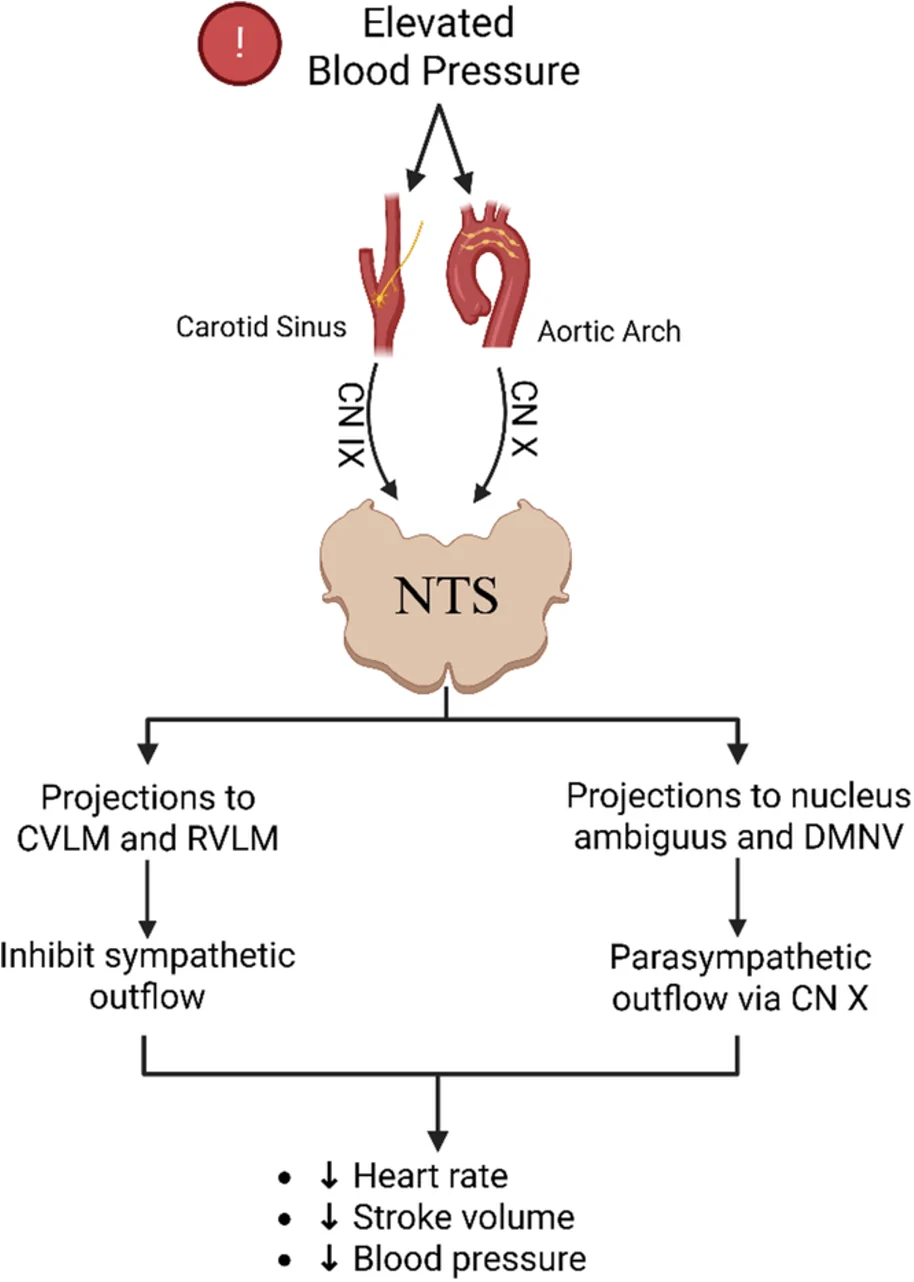
Neural regulation of blood pressure via baroreceptors reflexes. When blood pressure rises, stretch‐sensitive baroreceptors in the carotid sinus and aortic arch detect this change and send afferent signals via cranial nerves IX and X to the nucleus tractus solitarius (NTS) in the medulla. The NTS processes this input and acts on two pathways: it inhibits sympathetic outflow through projections to the caudal and rostral ventrolateral medulla (CVLM and RVLM) and simultaneously enhances parasympathetic output via projections to the nucleus ambiguus and dorsal motor nucleus of the vagus (DMNV). The resulting decrease in heart rate, stroke volume, and systemic vascular resistance restores blood pressure to normal levels. Dysfunction in this reflex results in sustained sympathetic activation, contributing to hypertension. Created with BioRender.com.

During hypotensive episodes, diminished baroreceptor activity leads to a reduction in parasympathetic tone and disinhibition of the RVLM. This culminates in amplified sympathetic efferent signaling through the intermediolateral cell column of the spinal cord, leading to increased heart rate and peripheral vascular resistance (Struthers and Dollery 1985). Notably, sympathetic stimulation of β_1_‐adrenergic receptors on juxtaglomerular cells induces renin secretion, thus engaging the RAAS‐axis to further modulate blood pressure (Patel et al. 2017). These mechanisms enable rapid and sustained adjustments to hemodynamic demands in response to internal and external stimuli (Figure 3).

**Figure 3:**
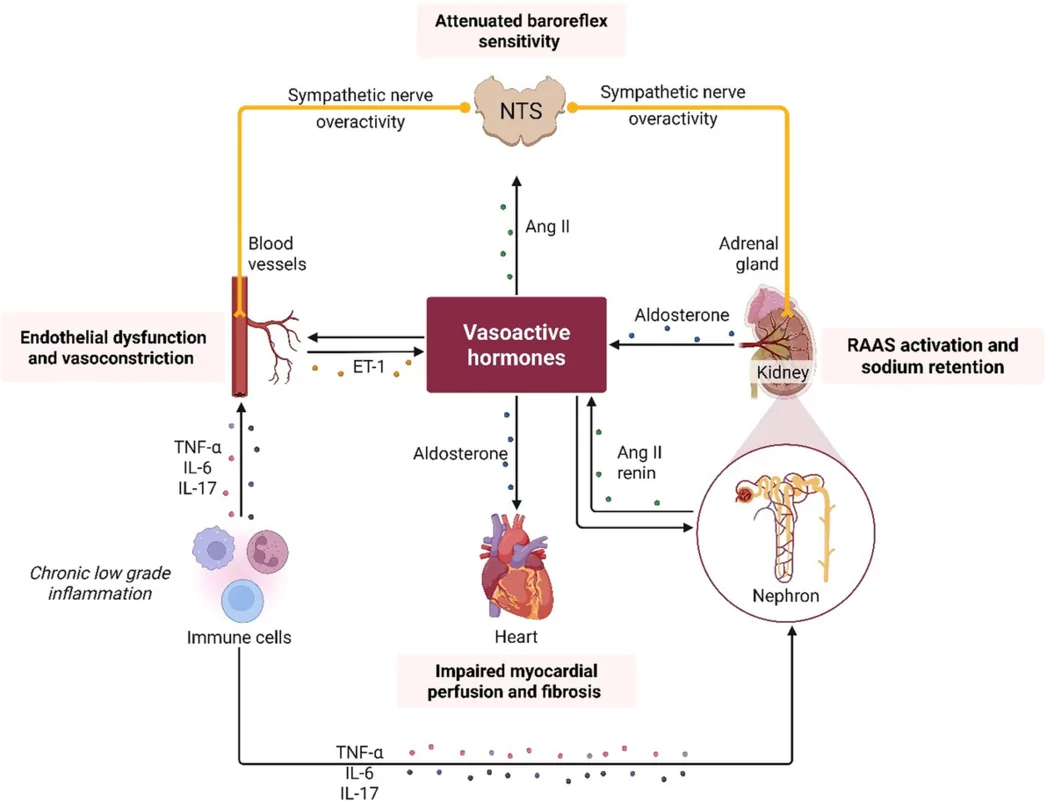
Interaction between neural, immune, and renal pathways in the pathogenesis of hypertension. Attenuated baroreflex sensitivity at the level of the nucleus tractus solitarius (NTS) contributes to sustained sympathetic nerve overactivity, which enhances neurohumoral signaling and promotes target‐organ dysfunction. Increased sympathetic drive stimulates renin–angiotensin–aldosterone system (RAAS) activation and renal sodium retention, leading to amplified production of vasoactive hormones such as angiotensin II, aldosterone, and endothelin‐1 (ET‐1). These mediators act on the vasculature, heart, and kidney, promoting endothelial dysfunction, vasoconstriction, myocardial fibrosis, and impaired renal pressure–natriuresis, respectively. Concurrently, chronic low‐grade inflammation, driven by cytokines including TNF‐α, IL‐6, and IL‐17, further exacerbates vascular and renal injury. Created with BioRender.com.

Sustained sympathetic overactivity or attenuated parasympathetic tone contributes substantially to the development of hypertension. Similar to RAAS dysregulation, this autonomic imbalance is influenced by various factors, including poor dietary habits, insulin resistance, circadian rhythm disruption, and microbiota dysbiosis (discussed in subsequent sections). Genetic factors also shape sympathetic nervous system activity; polymorphisms in genes encoding phosphoducin and neuropeptide Y receptors are associated with heightened sympathetic activity. Phosphoducin, a cytosolic protein expressed in the central nervous system and sympathetic ganglia, modulates G‐protein–mediated signaling by attenuating signal transduction at sympathetic synapses, while neuropeptide Y receptors contribute to vasoconstriction and sympathetic tone by regulating neurotransmitter release (Grassi 2021). Variants in these genes have been linked to increased blood pressure and exaggerated adrenergic responses, reinforcing the role of genetic predisposition in the development of neurogenic hypertension (Grassi 2021).

Manifesting as amplified sympathetic nerve activity (SNA) predominantly targeting the kidneys, heart, and vasculature, CNS dysregulation precipitates a cascade of hemodynamic, renal, and neurohumoral alterations conducive to persistent blood pressure elevation (Esler 2000). Notably, sympathetic overactivity appears early along the cardiovascular continuum and is detectable even in individuals with high‐normal or borderline blood pressure (Grassi and Drager 2024). In fact, the magnitude of sympathetic activation has been shown to parallel hypertension severity, suggesting that neuroadrenergic activation precedes overt hypertension rather than merely reflecting established disease (Grassi 1998). Within the CNS, crosstalk between RAAS and neural circuits is critical to hypertensive pathophysiology. Indeed, elevated central angiotensin II signaling engenders ROS production and NO depletion within the hypothalamic paraventricular nucleus (PVN), a key autonomic control hub (Esler 2000). This redox imbalance impairs inhibitory neurotransmission while enhancing excitatory inputs, rendering PVN neurons hyperexcitable and ultimately increasing sympathetic outflow to peripheral organs (Esler 2000). Moreover, oxidative stress attenuates baroreflex sensitivity, effectively shifting the set point toward elevated arterial pressure and perpetuating sympathetic overdrive (Esler 2000).

The pathological elevation in sympathetic tone is further exacerbated by proinflammatory cytokines such as interleukin‐1β (IL‐1β) and tumor necrosis factor‐alpha (TNF‐α). These cytokines are upregulated within the PVN in response to angiotensin II and oxidative stress, thereby amplifying sympathetic neuronal activation (Esler 2000). Additionally, peripheral afferent inputs contribute to the exaggerated central sympathetic drive. A key example is the renal afferent reflex, where mechanosensitive and chemosensitive fibers originating in the renal pelvis and interstitium transmit signals to the brainstem, ultimately modulating central sympathetic outflow (Seravalle and Grassi 2022). These afferents can be activated by local chemical stimuli, such as increased ROS, albumin overload, or intrarenal activation of RAAS, as well as mechanical signals like elevated renal interstitial pressure (Harrison et al. 2021). Together, these observations highlight the kidney–brain axis as a key contributor to hypertensive pathophysiology.

At effector sites, heightened SNA promotes a hypertensive milieu by potentiating renal sodium retention, vasoconstriction, and cardiac stimulation, with renal effects exerting the greatest influence (Seravalle and Grassi 2022). Simultaneously, α‐adrenergic stimulation in proximal tubular cells enhances sodium reabsorption, which expands extracellular fluid volume and increases preload, thereby contributing to elevated blood pressure. Experimental studies have validated these mechanisms and expanded the understanding of their interplay with other contributing factors. In a rabbit model of high‐fat diet–induced hypertension, renal SNA, mean arterial pressure (MAP), plasma leptin, and insulin were significantly elevated within 1 week of dietary intervention (Armitage et al. 2012). Even after dietary reversal, sympathetic tone and arterial pressure remained elevated, implying a feedforward loop involving persistent central activation, possibly via the PVN (Armitage et al. 2012).

Recent insights also expose a bidirectional interplay between inflammation and sympathetic nervous system overactivity (Caillon and Schiffrin 2016). Indeed, evidence shows that enhanced sympathetic drive not only triggers T‐lymphocyte activation but also amplifies vascular inflammation, possibly through desensitization of β_2_‐adrenoceptors on lymphocytes (Werdan 1998). While acute stimulation of these receptors typically exerts immunosuppressive effects, sustained sympathetic overactivity induces their desensitization and internalization, thereby blunting endogenous anti‐inflammatory control mechanisms (Werdan 1998). This maladaptation fosters excessive cytokine release, particularly interleukin‐6 (IL‐6) or TNF‐α from T‐cells or macrophages, respectively. Intriguingly, circulating levels of these pro‐inflammatory cytokines correlate positively with plasma norepinephrine concentrations, highlighting the reciprocal synergism between sympathetic activation and immune response (Grassi 2021). Collectively, these processes cultivate a proinflammatory vascular milieu, characterized by endothelial dysfunction, vascular remodeling, and increased arterial stiffness, key contributors to the progression and perpetuation of hypertension (Grassi 2021).

SNA amplification exerts effects that extend beyond the development of hypertension to include the promotion of hypertension‐mediated end‐organ damage (Quarti‐Trevano et al. 2023). Persistent sympathetic activation drives structural and functional injury across the cardiovascular system, contributing to arterial stiffness and left ventricular hypertrophy (LVH), which represent early manifestations of target‐organ involvement (Quarti‐Trevano et al. 2023). At the cardiac level, heightened sympathetic signaling, acting in concert with RAAS activation, increases preload and afterload, while β_1_‐adrenergic stimulation enhances intracellular Ca^2+^ mobilization in cardiomyocytes (Quarti‐Trevano et al. 2023). Although initially compensatory through augmentation of myocardial contractility, this response simultaneously increases myocardial oxygen demand, predisposing to maladaptive remodeling. Sustained neurohumoral activation establishes a self‐perpetuating cycle characterized by cardiomyocyte apoptosis, impaired myocardial perfusion, fibrosis, and progressive structural disorganization, ultimately culminating in myocardial dysfunction (Grassi et al. 2020). Clinical observations further demonstrate strong correlations between indices of sympathetic overactivity and increasing left ventricular mass, supporting a mechanistic link between neuroadrenergic drive and adverse cardiac remodeling (Grassi et al. 2020).

## 4 Activation of Inflammatory and Oxidative Stress Pathways

A paradigm shift has fundamentally transformed our understanding of hypertension, positioning immune system activation at the core of its pathogenesis and subsequent end‐organ injury (Rodriguez‐Iturbe et al. 2017). Accumulating experimental and clinical evidence demonstrates that chronic immune activation and inflammatory signaling represent key drivers of vascular dysfunction and blood pressure elevation (Aboukhater et al. 2023). Classical hypertensive stimuli, such as high salt intake and increased vascular stretch, provoke immune responses that perpetuate inflammation and oxidative stress across critical target organs, such as the kidney, vasculature, heart, and CNS (Rodriguez‐Iturbe et al. 2017). Importantly, these systems, CNS, RAAS, inflammation, and oxidative stress, do not function in isolation; rather, they engage in dynamic, bidirectional crosstalk, synergistically amplifying vascular dysfunction, arterial stiffness, and end‐organ damage (Harrison et al. 2021; Vaziri and Rodríguez‐Iturbe 2006).

At the molecular level, oxidative stress emerges from an imbalance favoring excessive production of ROS over antioxidant systems (Krzemińska et al. 2022). While ROS are essential for physiological signaling, such as vascular tone and immune defense, excessive and sustained ROS generation leads to cellular dysfunction and tissue injury, especially in vascular and renal tissues (Krzemińska et al. 2022). In hypertension, the principal sources of ROS include NOX isoforms, uncoupled eNOS, mitochondria, and xanthine oxidase (Figure 4) (Z. Zhang et al. 2022). Among these, the NOX family enzymes, particularly NOX1, NOX2, and NOX4, are dominant contributors and are activated by angiotensin II, endothelin‐1 (ET‐1), aldosterone, and mechanical stress, leading to the generation of superoxide and hydrogen peroxide (Z. Zhang et al. 2022). In turn, these ROS contribute not only to direct tissue damage but also to impaired NO signaling, as superoxide rapidly scavenges NO, forming the highly reactive and deleterious peroxynitrite (Z. Zhang et al. 2022).

**Figure 4:**
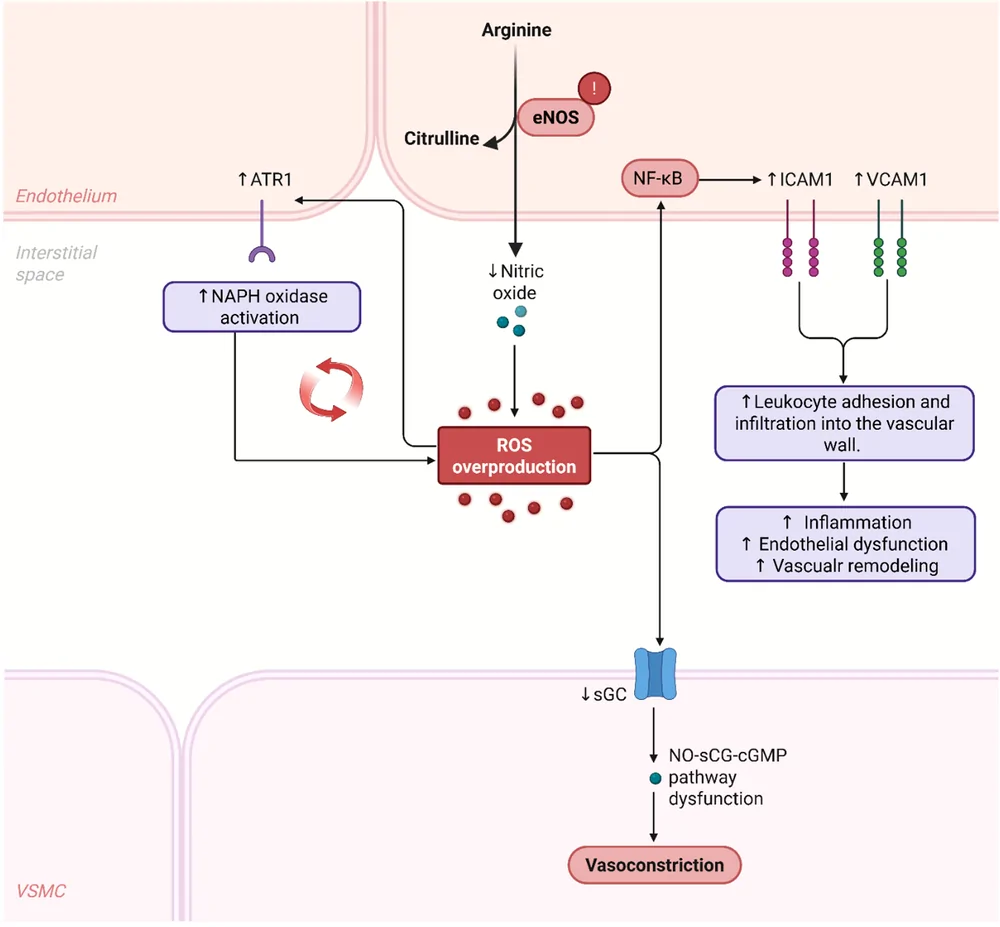
Role of oxidative stress in the pathogenesis of hypertension. Uncoupling of endothelial nitric oxide synthase (eNOS) reduces nitric oxide (NO) production and increases superoxide generation, leading to the overproduction of reactive oxygen species (ROS). In turn, excess ROS enhance Angiotensin II Type 1 Receptor (AT1R) signaling and promote NADPH oxidase activation, further increasing ROS levels in a pathological feed‐forward loop. This ROS overload ultimately impairs the Nitric Oxide–Soluble Guanylyl Cyclase–Cyclic Guanosine Monophosphate (NO–sGC–cGMP) pathway, disrupting vascular smooth muscle relaxation and sustaining vasoconstriction. These effects collectively elevate vascular resistance and contribute to the development of hypertension. Simultaneously, ROS activate NF‐κB signaling, upregulating endothelial adhesion molecules such as ICAM‐1 and VCAM‐1, thereby promoting leukocyte adhesion and infiltration into the vascular wall. This inflammatory cascade further exacerbates endothelial dysfunction, promotes vascular remodeling, and perpetuates the hypertensive state. Created with BioRender.com.

The resultant oxidative insult precipitates endothelial dysfunction, a hallmark of hypertension, characterized by impaired vasodilation, phenotypic switch of vascular smooth muscle cell (VSMC), and increased vascular tone, all contributing to elevated systemic vascular resistance (Vaziri and Rodríguez‐Iturbe 2006). Chronic oxidative stress further drives endothelial injury, endothelial‐to‐mesenchymal transition, and maladaptive remodeling of the vascular wall, thereby linking redox imbalance to the structural arterial changes underlying hypertension. ROS further activate redox‐sensitive transcription factors such as nuclear factor‐kappa B (NF‐κB), activator protein‐1 (AP‐1), and mitogen‐activated protein kinases (MAPKs), which in turn upregulate pro‐inflammatory genes (Vaziri and Rodríguez‐Iturbe 2006). Particularly, NF‐κB activation within the endothelium induces the expression of cytokines, including TNF‐α, IL‐6 and IL‐1β, and chemokines, such as chemokine (C‐C motif) ligand 2 (CCL2) and CCL5. Additionally, NF‐κB promotes the expression of adhesion molecules, such as intercellular adhesion molecule‐1 (ICAM‐1) and vascular cell adhesion molecule‐1 (VCAM‐1), thereby facilitating leukocyte adhesion and infiltration into the vascular wall (Figure 4) (Dinh et al. 2014; Polla and Cossarizza 1996). This cascade converts a transient oxidative insult into chronic vascular inflammation (Vaziri and Rodríguez‐Iturbe 2006). The persistent presence of immune cells sustains ROS generation and vascular remodeling, establishing a self‐amplifying circuit that exacerbates hypertension (Vaziri and Rodríguez‐Iturbe 2006).

ROS further promote NF‐κB activation in kidneys, particularly in glomerular mesangial cells, tubular epithelial cells, and podocytes (Ren et al. 2023). In mesangial cells, NF‐κB activation drives proliferation, matrix expansion, and loss of mesangial architecture, contributing to glomerulosclerosis (Ren et al. 2023). In renal tubular epithelial cells, NF‐κB signaling upregulates pro‐inflammatory cytokines and chemokines, promotes tubular cell apoptosis and pyroptosis, and drives tubulointerstitial inflammation and fibrosis (Ren et al. 2023). Beyond these structural consequences, NF‐κB activation may also disrupt renal sodium handling through its interplay with inflammatory pathways (Ahmad et al. 2023). Specifically, NF‐κB provides the priming signal for NLRP3 inflammasome activation by inducing the expression of NLRP3 and pro‐IL‐1β (Ahmad et al. 2023). Subsequent inflammasome activation promotes renal inflammation and impairs sodium excretion, thereby favoring sodium retention and ultimately contributing to hypertension (Ahmad et al. 2023).

Intriguingly, NF‐κB activation across renal, vascular, and cardiac tissues is not driven solely by ROS but can also occur downstream of Toll‐like receptor 4 (TLR4) activation (Küper et al. 2012). TLR4 is activated by pathogen‐associated molecular patterns, most notably lipopolysaccharide (LPS), as well as endogenous damage‐associated molecular patterns released during cellular injury, including high‐mobility group box 1 (HMGB1), heat shock proteins, and uric acid crystals (H. Wu et al. 2007). Several mechanisms implicated in hypertension can also converge on this pathway. For instance, impaired intestinal barrier integrity in gut dysbiosis facilitates the systemic translocation of bacterial products such as LPS, providing a potential stimulus for TLR4 activation (O'Donnell et al. 2023). In parallel, activation of RAAS further reinforces TLR4 signaling, as angiotensin II has been shown to increase TLR4 expression and promote TLR4‐dependent oxidative and inflammatory responses (Nakashima et al. 2015). Consequently, ligand recognition by TLR4 promotes receptor dimerization and engagement of its co‐receptors, followed by recruitment of the adaptor protein myeloid differentiation primary response 88 (MyD88) (H. Wu et al. 2007). This cascade ultimately promotes IκB phosphorylation and degradation, releasing NF‐κB from its inhibitory complex and enabling its nuclear translocation and subsequent transcription of pro‐inflammatory genes (Küper et al. 2012).

ROS and TLR4 signaling also activate MAPK pathways, including ERK1/2, JNK, and p38, which contribute to vascular and renal injury in hypertension (Sahu and Rawal 2024). Within the vasculature, JNK and p38 MAPK cascades promote VSMC proliferation, migration, and phenotypic switching, thereby contributing to maladaptive vascular remodeling and arterial stiffening (Sahu and Rawal 2024). Similarly, MAPK activation within the renal parenchyma promotes profibrotic responses, including tubular epithelial cell dysfunction and increased extracellular matrix production, ultimately contributing to tubulointerstitial fibrosis (Lenhard et al. 2003). Accordingly, dysregulated MAPK has emerged as a potential therapeutic target, with several medications and naturally derived compounds being investigated for their ability to modulate MAPK signaling and thereby attenuating hypertension (Lenhard et al. 2003; Sahu and Rawal 2024)

Innate immune cells, mainly macrophages, are critical agents in hypertensive pathology. These highly plastic and heterogeneous cells infiltrate hypertensive target organs, including the kidney, vasculature, and heart, where they orchestrate inflammatory injury (Figure 5) (Harrison and Patrick 2024). Upon recruitment into these tissues, macrophages differentiate into two distinct functional phenotypes broadly categorized as proinflammatory M1 macrophages and anti‐inflammatory M2 macrophages (Ley 2017). In the context of hypertension, M1 macrophages are the predominant infiltrating subtype. They produce high levels of proinflammatory cytokines, including TNF‐α, IL‐1β and IL‐6, ROS, and matrix metalloproteinases, which directly target VSMCs, renal tubular cells, and resident immune cells, ultimately contributing to endothelial dysfunction, vascular remodeling, and sodium retention (Harrison and Patrick 2024). Additionally, the accumulation of M1 macrophages in the vascular adventitia and renal interstitium has been associated with elevated NADPH oxidase activity, impaired vasodilation, and pressure‐natriuresis dysregulation (Harrison and Patrick 2024). Thus, macrophage‐driven inflammation signified a critical pathological axis in hypertensive pathophysiology.

**Figure 5:**
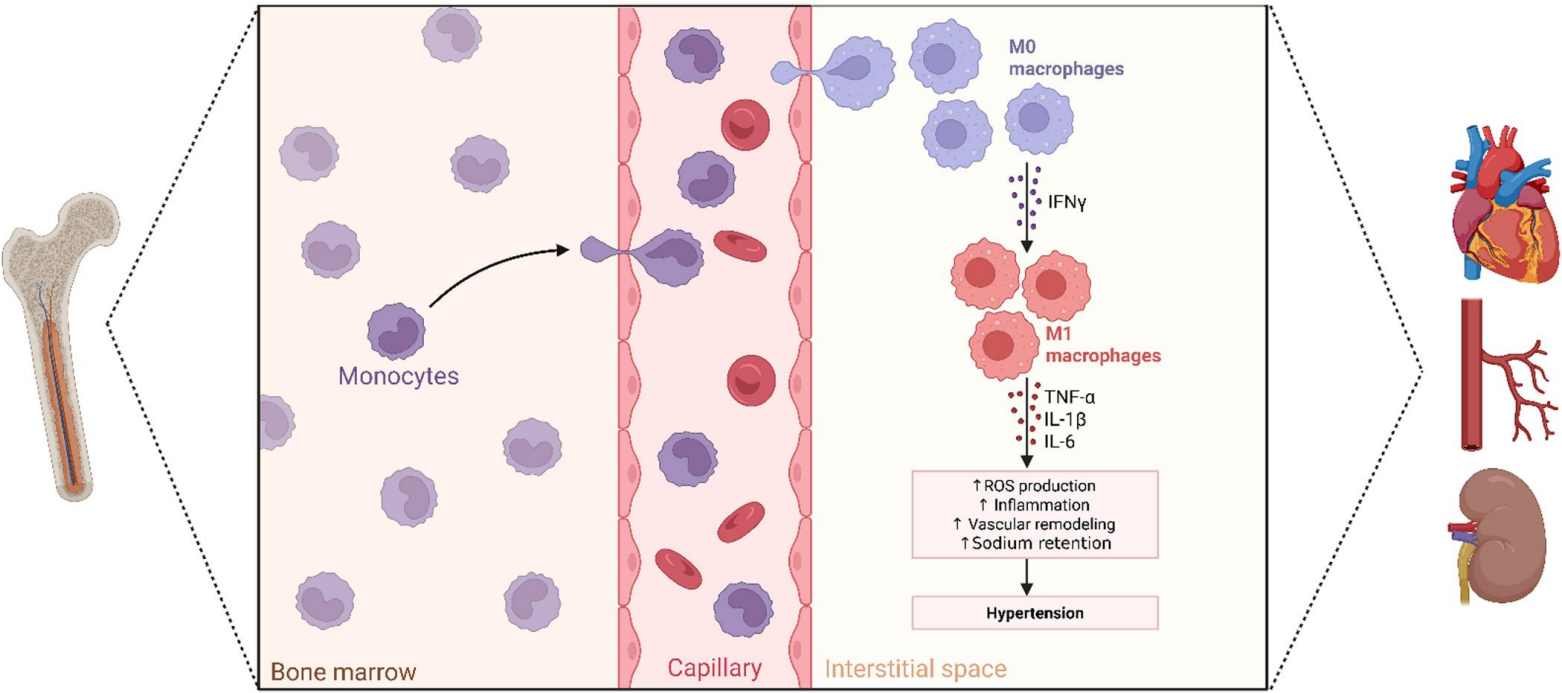
Immune activation and macrophage polarization in hypertension. Monocytes originating from the bone marrow migrate into the circulation and infiltrate hypertensive target organs, including the kidney, vasculature, and heart. Upon entering the interstitial space, these monocytes adopt a resting macrophage state known as M0. In response to pro‐inflammatory signals such as interferon‐gamma (IFN‐γ), M0 macrophages become polarized into pro‐inflammatory M1 macrophages. These M1 cells secrete cytokines including tumor necrosis factor‐alpha (TNF‐α), interleukin‐1β (IL‐1β), and interleukin‐6 (IL‐6), which promote reactive oxygen species (ROS) production, amplify local inflammation, and contribute to vascular remodeling and sodium retention. Collectively, these immune‐mediated processes disrupt vascular and renal homeostasis, ultimately driving the development and maintenance of hypertension. Created with BioRender.com.

Dendritic cells (DCs) are professional antigen‐presenting cells within the innate immune system and also appear to play a role in hypertension (Van Beusecum et al. 2022). Studies demonstrate that DCs from hypertensive animals, when transferred to normotensive hosts, induce heightened susceptibility to hypertensive stimuli, illustrating their preactivated pathogenic state (Chiasson et al. 2018). Mechanistically, DCs produce and accumulate isolevuglandins (isoLGs), highly reactive lipid peroxidation products which covalently modify self‐proteins into neoantigens (Van Beusecum et al. 2022). These neoantigens are presented by DCs to T‐cells, facilitating activation and expansion of adaptive immune responses (Van Beusecum et al. 2022). This bridge between innate and adaptive immunity established a feed‐forward circuit that sustains blood pressure elevation and inflammatory tissue injury.

The adaptive immune system is indispensable for sustaining and amplifying hypertensive pathology (Harrison et al. 2021). Evidence for T‐cell involvement in hypertension emerged from studies utilizing mice that lack mature T‐ and B‐cells (Guzik et al. 2007). These mice exhibited blunted hypertensive responses to angiotensin II and salt challenges. However, these effects were reversed by adoptive transfer of T‐cells, confirming a causal role for T‐cells in the development of hypertension (Guzik et al. 2007). Notably, proinflammatory T‐cell subsets, including T‐helper 1 (Th‐1), T‐helper 17 (Th‐17), and cytotoxic CD8^+^ T‐cells accumulate within the vasculature, kidney, and perivascular adipose tissue, secreting cytokines (IFN‐γ and TNF‐α, IL‐17A) and cytotoxic mediators (perforin and granzymes) that exacerbate endothelial dysfunction, vascular remodeling, and renal injury (Harrison et al. 2010). IL‐17A, in particular, promotes superoxide production, increases expression of adhesion molecules, and reduces NO bioavailability, all of which disrupt blood pressure homeostasis, eventually precipitating hypertension (Harrison et al. 2010). Evidence also implicated B cells in driving elevated blood pressure and promoting vascular inflammation. Mechanistically, these effects are linked to B‐cell production of autoantibodies against AT_1_R and adrenergic receptors. These autoantibodies activate pathogenic pathways that facilitate vascular remodeling and cardiac dysfunction (Mikolajczyk and Guzik 2019). Intriguingly, such autoantibodies have been detected in both hypertensive patients and spontaneously hypertensive mice, where they have been suggested to promote vascular remodeling and end‐organ damage (Mikolajczyk and Guzik 2019). These findings indicate that B‐cell–mediated humoral responses represent a potentially underrecognized axis in the immune‐driven pathogenesis of hypertension.

Beyond these vascular and systemic effects, innate and adaptive immune activation extends to the kidneys, where it induces structural and functional alterations that contribute to the development of hypertension (Figure 6). Within the renal parenchyma, tubular epithelial cells release IL‐1β, a cytokine that recruits and activates renal macrophages. This, in turn, potentiates the expression of the epithelial sodium channel (ENaC), ultimately promoting salt‐sensitive hypertension (Veiras et al. 2022). In parallel, macrophage‐derived IL‐1β acts through IL‐1 receptor on myeloid cells to limit their maturation into NO‐producing macrophages (J. Zhang et al. 2016). The resulting reduction in macrophage‐derived NO, which normally suppresses Na^+^‐K^+^−2Cl^‐^ cotransporter (NKCC2) activity, promotes NKCC2‐mediated sodium reabsorption in thick ascending limbs, thereby exacerbating hypertension (J. Zhang et al. 2016).

**Figure 6:**
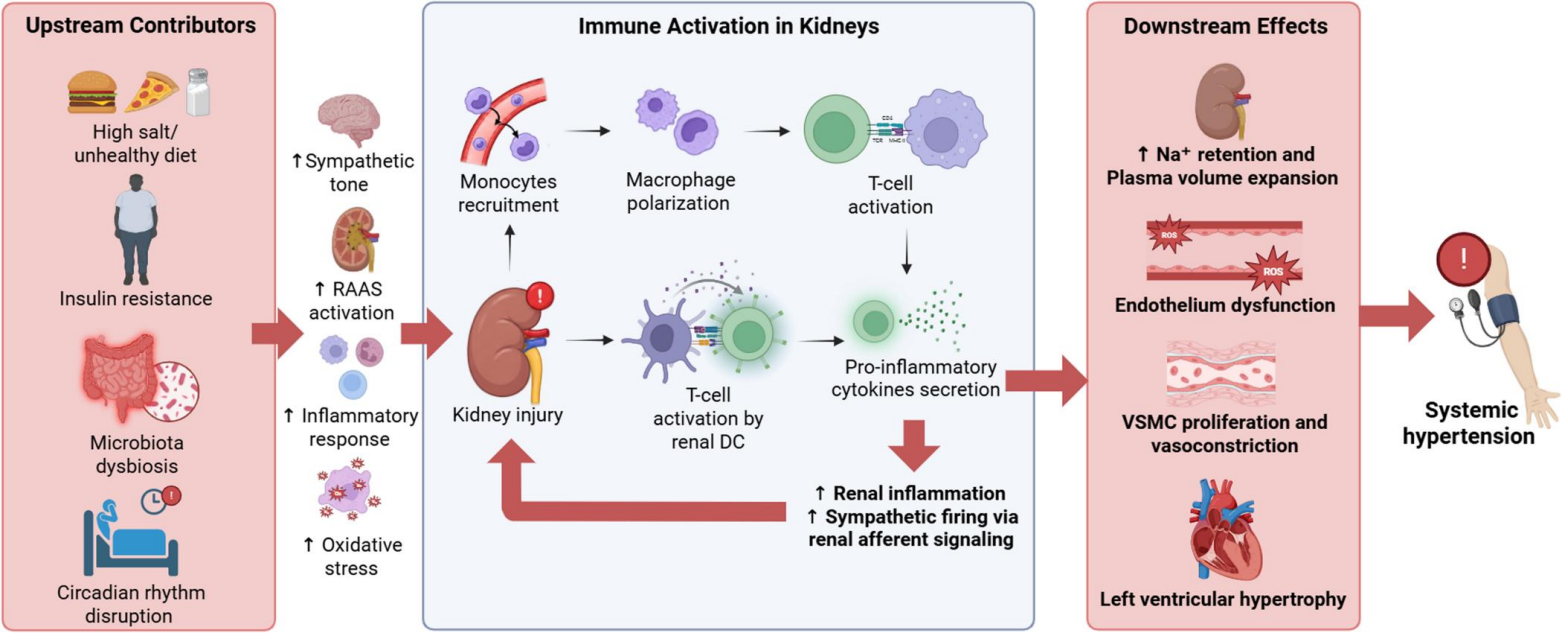
Integrated dietary, neural, renal, and immune mechanisms contributing to systemic hypertension. Dietary and metabolic disturbances, including high salt intake, insulin resistance, microbiota dysbiosis, and circadian rhythm disruption, converge on increased sympathetic activity, RAAS activation, inflammation, and oxidative stress, promoting renal injury and immune activation. Within the kidney, recruitment of circulating monocytes and their polarization into macrophages, together with DC–mediated T‐cell activation, amplifies local inflammation through pro‐inflammatory cytokine release. This renal immune response establishes a feed‐forward cycle of kidney injury, inflammation, and enhanced sympathetic signaling. The resulting sodium retention, endothelial dysfunction, VSMC proliferation and vasoconstriction, and cardiac remodeling collectively increase circulating volume and vascular resistance, ultimately driving systemic hypertension. Created with BioRender.com.

Furthermore, renal DCs contribute to hypertension by bridging innate and adaptive immune responses (Benson et al. 2023). DC‐primed T‐cells migrate into renal parenchyma, where they release TNF‐α, IFN‐γ, and IL‐17A, thus enhancing tubular sodium transport and promoting renal vasoconstriction, ultimately exacerbating sodium and fluid retention (Benson et al. 2023). Additionally, infiltrating CD8^+^ T‐cells can directly alter tubular sodium handling within the distal convoluted tubule, thereby promoting NADPH oxidase‐dependent ROS production and subsequent Src kinase activation (Liu et al. 2017). This activation enhances the activity of the Na^+^‐Cl^−^ cotransporter (NCC), potassium channel Kir4.1, and the chloride channel ClC‐K, collectively favoring sodium‐chloride reabsorption (Liu et al. 2017). Similarly, CD4^+^ T‐helper subsets, particularly Th‐17, further reinforce this response through IL‐17A secretion, which enhances renal sodium transporter activity and promotes renal injury (Lu and Crowley 2020). Collectively, these mechanisms establish the kidney as a key site where innate and adaptive immune responses converge to drive the pathogenesis of hypertension.

Clinical evidence further supports inflammatory activation in hypertension. Multiple population‐based studies have consistently shown that hypertensive individuals exhibit substantially higher circulating levels of inflammatory biomarkers, including C‐reactive protein (CRP), IL‐6 and TNF‐α, compared with normotensive individuals, indicating the presence of a chronic low‐grade inflammatory state in hypertension (Amponsah‐Offeh et al. 2023; Barati Boldaji et al. 2020). Moreover, in a cross‐sectional study, participants with higher cytokine quartiles demonstrated approximately two‐ to three‐fold greater prevalence of hypertension compared with those in the lowest quartile, further supporting a clinically relevant link between inflammatory biomarker elevation and blood pressure status (Bautista et al. 2005). Intriguingly, a meta‐analysis of 32 randomized controlled trials demonstrated that RAAS inhibition, particularly with ACE inhibitors, significantly reduces circulating CRP, IL‐6, and TNF‐α levels, suggesting that the clinical benefits of ACE inhibitors may be attributed not only to RAAS inhibition, but also to their anti‐inflammatory effects (Awad et al. 2022).

Additionally, interventions that attenuate oxidative stress and systemic inflammation, such as regular physical activity, weight reduction, and dietary optimization, are consistently associated with improved blood pressure control (Hayes et al. 2014). In contrast, lifestyle factors and metabolic conditions that increase oxidative burden, including smoking, dyslipidemia, obesity, and insulin resistance, promote endothelial dysfunction and vascular inflammation, thereby contributing to the development and progression of hypertension (Amponsah‐Offeh et al. 2023). Beyond conventional antihypertensive management, experimental work further highlights novel immune‐targeted strategies, such as the endogenous anti‐inflammatory protein developmental endothelial locus‐1 (DEL‐1), which reduces immune cell recruitment, improves endothelial function, and attenuates vascular and cardiac remodeling in hypertensive models, suggesting potential future translational applications (Henrion 2022). Emerging evidence also suggests that direct immune modulation may represent a promising adjunctive strategy in hypertension. Experimental and early clinical studies indicate that several immunomodulatory agents, including TNF‐α inhibitors, mycophenolate mofetil, methotrexate, hydroxychloroquine, and selected cytokine‐targeted therapies, can reduce blood pressure and attenuate hypertension‐mediated organ damage, although most studies were not primarily designed to evaluate blood pressure outcomes (Murray et al. 2021). Among these agents, TNF‐α inhibitors have shown the most consistent blood pressure reduction effect, with findings derived primarily from patients with rheumatoid arthritis and other chronic inflammatory diseases, reinforcing a mechanistic link between cytokine signaling and vascular dysfunction (Sandoo et al. 2011; Yoshida et al. 2014; Zhao et al. 2015). Proposed mechanisms include attenuation of systemic inflammation, improved endothelial nitric oxide bioavailability, and reduced oxidative stress, which together may lower peripheral vascular resistance and contribute to improved blood pressure control (Yoshida et al. 2014). More targeted cytokine approaches, however, have yielded less consistent findings (R Muralitharan et al. 2024). In the Canakinumab Anti‐inflammatory Thrombosis Outcomes Study (CANTOS) trial, IL‐1β inhibition with canakinumab did not reduce incident hypertension overall, although reductions in blood pressure were observed among participants with baseline systolic blood pressure ≥ 130 mmHg (Ridker et al. 2017). Likewise, IL‐17A inhibition with secukinumab failed to significantly reduce blood pressure in the FIXTURE trial, although interpretation was limited by the largely normotensive study population (Langley et al. 2014; Thaci et al. 2016). Beyond cytokine‐directed agents, inflammasome‐targeted approaches are also advancing clinically. The NLRP3 inhibitor dapansutrile (OLT1177) has progressed into clinical development for inflammatory and cardiometabolic conditions, yet a clear antihypertensive effect has not been established (Tluli et al. 2026). Collectively, these findings provide proof‐of‐concept that modulation of immune pathways may influence blood pressure, but their translation into antihypertensive therapy remains challenging.

## 5 Dietary Factors

Dietary factors exert a profound impact on the regulation of blood pressure, and are critical determinants in both the prevention and management of hypertension. Evidence shows that a balanced nutrition, particularly diets abundant in fruits, vegetables, and dietary potassium, but restricted in sodium, red meat, and saturated fat consistently demonstrate cardiovascular benefits and reduce risk of cardiometabolic diseases including hypertension (Ndanuko et al. 2016). These findings form the foundation of the Dietary Approaches to Stop Hypertension (DASH) diet, which assimilates evidence‐based nutritional principles into a cohesive framework intended for wide clinical and public health application (Ozemek et al. 2018).

Development and widespread adoption of the DASH diet represent a systematic and pragmatic response to the need for nonpharmacologic interventions in hypertension (Figure 7). It emphasizes generous servings of fruits, vegetables, whole grains, and low‐fat dairy products, alongside lean sources of proteins such as poultry, fish, and legumes. This composition ensures a rich intake of potassium, magnesium, calcium, and fiber, all of which are implicated in vascular homeostasis (Challa et al. 2025). The reduction of red meat, saturated fat, cholesterol, and refined sugar further supports cardiovascular health and aligns with contemporary dietary guidelines (Challa et al. 2025). Notably, implementation of the DASH diet has been associated with improved lipid profiles and enhanced insulin sensitivity, both of which contribute to blood pressure. Moreover, this diet can reduce SBP by approximately 5–6 mmHg and DBP by 3 mmHg in normotensive individuals, with effects magnified in hypertensive participants, where reductions of up to 11 mmHg in SBP and 6 mmHg in DBP have been observed in as little as 8 weeks (Appel et al. 1997). These reductions translate to measurable decreases in risk for stroke, myocardial infarction, type 2 diabetes, and obesity, and are substantiated by a 13% reduction in the 10‐year cardiovascular risk as assessed by the Framingham risk score (Challa et al. 2025; Siervo et al. 2015). Thus, the DASH diet has cemented its role as a cornerstone of non‐pharmacologic hypertension management. Beyond global dietary patterns, a growing body of work highlights bioactive phytochemicals, particularly polyphenolic flavonoids and garlic‐derived compounds, as modulators of endothelial function, oxidative stress, and vascular tone that may confer additional vasculoprotecrive and antihypertensive benefits (Mansour et al. 2024).

**Figure 7:**
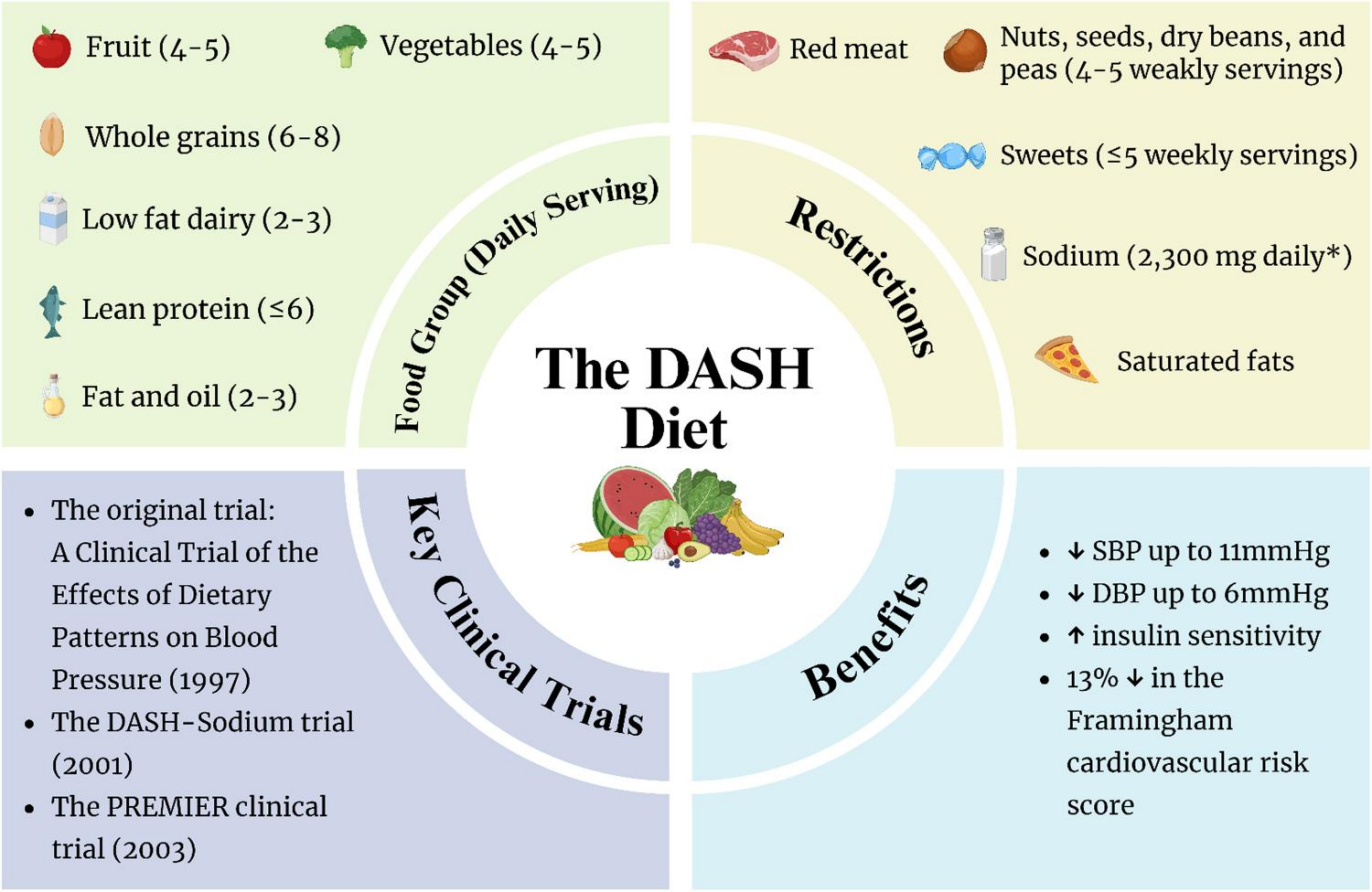
DASH dietary pattern's effects on blood pressure regulation. The DASH diet is rich in fruits, vegetables, whole grains, and low‐fat dairy while limiting sodium, red meat, and sweets. Clinical trials, including the original DASH study, the DASH‐Sodium trial, and the PREMIER trial, demonstrated significant reductions in systolic and diastolic blood pressure, improved insulin sensitivity, and reduced long‐term cardiovascular risk. *Intensive version of the DASH diet limits sodium intake to 1,500 mg per day as it results in greater reductions in blood pressure, particularly among individuals with hypertension or at elevated cardiovascular risk. Created with BioRender.com.

Reduction of dietary sodium, primarily sourced from table salt and processed foods, further augments the antihypertensive efficacy of the DASH diet. The landmark DASH‐Sodium trial systematically investigated the effects of graded sodium reduction in participants adhering to either a DASH or traditional Western diet. By adjusting sodium intake across high, intermediate, and low levels, the study found incremental SBP reductions in both dietary arms, with the most pronounced benefit observed under low‐sodium conditions: the combination of the DASH diet with sodium intake restricted to approximately 1500 mg/day produced reductions of 7.1 mmHg in normotensives and 11.5 mmHg in hypertensive individuals compared to a high‐sodium Western diet (Sacks et al. 2001). These findings clearly implicate sodium as a modifiable risk factor, reinforcing the necessity of sodium restriction as a core strategy for blood pressure control.

In contrast to sodium's pressor effects, dietary potassium appears to have a protective effect against hypertension, especially in the context of high sodium consumption. A meta‐analysis of 33 randomized controlled trials demonstrated that potassium supplementation alone significantly reduced mean SBP and DBP by 3.11 and 1.97 mmHg, respectively (Whelton 1997). Mechanistically, dietary potassium improves renal perfusion and inhibits renal sodium transporters in the proximal tubule and loop of Henle, thereby promoting natriuresis and reducing extracellular fluid volume (Poulsen and Fenton 2019). Simultaneously, elevated potassium directly suppresses renin release and subsequently reduces angiotensin II and aldosterone levels (M. X. Wang et al. 2018). The net effect is a reduction in vascular resistance and arterial stiffness, processes further aided by potassium‐induced enhancement of endothelial NO production and diminished sensitivity to vasoconstrictors (D'Elia et al. 2023; Poulsen and Fenton 2019).

At the cellular level, both sodium and potassium play central roles in regulating blood pressure, with alterations in their homeostasis directly contributing to the development and maintenance of hypertension. In VSMCs, increased dietary sodium results in raise in intracellular calcium, the primary mediator of VSMCs contraction, by altering Na^+^‐transporters such as the Na^+^/Ca^2+^ exchanger and Na^+^/H^+^ exchanger (Bkaily et al. 2021). This promotes stronger vasoconstriction and increased vascular tone, ultimately leading to elevated peripheral vascular resistance and contributing to the development of hypertension (Bkaily et al. 2021). Excess extracellular sodium also disrupts vascular homeostasis through direct molecular effects that extend beyond volume expansion. At the endothelial surface, the glycocalyx forms a negatively charged barrier composed of heparan sulfate proteoglycans and hyaluronan that transiently binds circulating sodium ions, limiting their direct access to endothelial cells (Gaudette et al. 2020). This sodium‐buffering function preserves endothelial mechanotransduction, the conversion of shear stress generated by blood flow into intracellular biochemical signals, particularly those activating endothelial NO synthase, thereby maintaining NO–mediated vasodilation (Gaudette et al. 2020). Chronic sodium excess overwhelms this buffering capacity, leading to degradation and shedding of glycocalyx components through activation of proteolytic and glycosaminoglycan‐cleaving enzymes (Sembajwe et al. 2023). Loss of glycocalyx integrity permits increased sodium penetration toward the endothelial membrane, impairs shear stress signaling, and ultimately reduces NO bioavailability (Sembajwe et al. 2023). These sodium‐induced alterations collectively shift the vascular phenotype toward vasoconstriction and reduced arterial compliance, creating a permissive environment for sustained blood pressure elevation.

In contrast, potassium counteracts sodium‐driven pressor mechanisms, such that inadequate potassium intake markedly amplifies the hypertensive effects of sodium excess. At the vascular level, adequate extracellular potassium concentration promotes endothelium‐dependent vasodilation by stimulating Na^+^/K^+^‐ATPase activity and activating potassium channels, leading to endothelial hyperpolarization (Houston 2011). This hyperpolarization is transmitted to adjacent VSMCs, stabilizing membrane potential at more negative values, reducing the activation of voltage‐gated calcium channels, and thereby suppressing calcium‐dependent actin–myosin cross‐bridge formation. The net effect is reduced vascular tone and peripheral resistance (Houston 2011). In parallel, potassium also exerts a dominant antihypertensive renal effect through the so‐called potassium switch, whereby it alters distal convoluted tubule membrane voltage and raises intracellular chloride concentration. In turn, this inhibits the with‐no‐lysine (WNK) kinase signaling cascade and downregulates the thiazide‐sensitive NCC, independent of aldosterone (Chan et al. 2024). This suppresses sodium reabsorption, enhancing natriuresis and ultimately reducing extracellular volume and blood pressure (Geleijnse et al. 2003). Beyond these primary mechanisms, potassium further attenuates vasoconstrictive responsiveness to norepinephrine and angiotensin II, enhances Na^+^/K^+^‐ATPase activity, and modulates baroreceptor sensitivity, collectively buffering the deleterious vascular and renal consequences of sodium excess (Chan et al. 2024). Together, these cellular mechanisms position potassium as a central determinant of blood pressure regulation rather than a secondary dietary factor.

The quality and composition of dietary fat represent additional modifiable elements with substantial impact on hypertension risk. Saturated fats, prevalent in red meat, butter, and full‐fat dairy, are detrimental to vascular health, as they promote oxidative stress, inhibit NO synthesis, and activate proinflammatory signaling, leading to increased vascular tone and arterial stiffness (Nestel 2019). Conversely, diets rich in unsaturated fats, notably polyunsaturated and omega‐3 fatty acids from plant oils, nuts, seeds, and fatty fish, foster endothelial health by supporting nitric oxidNO bioavailability, attenuating inflammation, and reducing oxidative burden (Nestel 2019). The Mediterranean dietary pattern exemplifies the beneficial effects of favoring unsaturated over saturated fats and has been consistently associated with lower hypertension incidence and improved vascular outcomes (Ozemek et al. 2018). Together, this highlights that dietary fat quality, rather than total fat intake, is a critical determinant in blood pressure regulation and hypertension risk.

## 6 Salt Sensitivity

Salt sensitivity embodies a distinct physiological phenomenon wherein an individual's blood pressure fluctuates significantly in response to changes in dietary sodium intake, a trait with profound implications for cardiovascular health (Balafa and Kalaitzidis 2021). Those classified as salt‐sensitive exhibit notable elevations in blood pressure following sodium loading, and marked reductions with sodium restriction, whereas salt‐resistant individuals maintain relatively stable blood pressure irrespective of sodium intake (Grillo et al. 2019). Landmark renal transplantation studies in genetically characterized murine models have solidified the kidney's central role, demonstrating that recipients of salt‐sensitive kidneys develop hypertension, thereby highlighting the importance of genotype on renal sodium handling and long‐term blood pressure regulation (Dahl et al. 1972). The clinical significance of salt sensitivity extends beyond its modifying effect on blood pressure, as it independently predicts heightened risk of cardiovascular events and mortality even among normotensive individuals (Weinberger 2002). Indeed, longitudinal studies reveal that normotensive but salt‐sensitive subjects have mortality curves akin to hypertensive patients, underscoring the prognostic necessity to identify this trait early for preventive interventions (Weinberger 2002). Recognizing salt sensitivity as an independent risk factor urges incorporation of tailored dietary and therapeutic approaches to reduce adverse cardiovascular outcomes.

A multitude of genetic variants underpin salt sensitivity, modulating renal sodium handling and vascular reactivity (Sanada et al. 2011). Among them, polymorphisms in the α‐adducin 1 (ADD1) and G protein–coupled receptor kinase 4 (GRK4) are consistently associated with exaggerated blood pressure elevations secondary to salt loading. The ADD1 protein modulates Na^+^/K^+^‐ATPase activity and thereby influences proximal tubular sodium reabsorption, while GRK4 orchestrates dopamine receptor desensitization, thereby disrupting natriuretic signaling in the kidney (Sanada et al. 2011). Mechanistically, increased dietary sodium expands extracellular fluid volume, which augments venous return and end‐diastolic filling, ultimately raising stroke volume and cardiac output, transiently raising arterial pressure (Kurtz et al. 2017). In salt‐resistant individuals, pressure natriuresis efficiently restores fluid balance by promoting sodium and water excretion. However, salt‐sensitive individuals exhibit blunted natriuretic and compensatory vascular responses, which then lead to sustained sodium retention, persistent volume overload, and progression to chronic hypertension (Castiglioni et al. 2011). Causative mechanisms extend to inappropriate or paradoxical activation of intrarenal renin–angiotensin system and enhanced oxidative stress, further exacerbating end‐organ damage. Collectively, these maladaptive renal and vascular processes anchor salt sensitivity as a key pathophysiological driver.

Intriguingly, sodium consumption in salt‐sensitive individuals initiates a two‐phase process, in which the initial volume‐driven rise in blood pressure is followed by a secondary immune‐mediated amplification phase (Vogt et al. 2023). In murine models of salt‐sensitive hypertension, particularly Dahl salt‐sensitive rats, impaired renal autoregulatory capacity allows transmission of elevated perfusion pressure to the intrarenal microvasculature, precipitating subtle but progressive renal tissue injury (Evans et al. 2017). This injury promotes the formation or exposure of antigens and neoantigens within the renal parenchyma, facilitating antigen presentation by resident DCs and monocyte/macrophage populations and triggering activation of adaptive immunity through classical T‐cell receptor–dependent pathways (Evans et al. 2017).

Beyond this indirect mechanism, sodium also exerts direct immunomodulatory effects. Increased extracellular sodium has been shown to activate DCs via amiloride‐sensitive channels, enhancing ROS generation and the formation of immunogenic isoketal‐modified proteins that further stimulate T‐cell activation (Vogt et al. 2023). Moreover, elevated sodium concentrations promote polarization of naïve T cells toward a pro‐inflammatory Th‐17 phenotype via serum and glucocorticoid‐regulated kinase‐1 (SGK1)‐dependent signaling, a process that may be further reinforced by salt‐induced alterations in the gut microbiome (Girardin et al. 1980). Consequently, macrophages, CD4^+^ T helper cells, CD8^+^ cytotoxic T cells, and B cells infiltrate the renal interstitium, particularly around damaged tubules and blood vessels, mirroring histological findings observed in kidney biopsies from patients with hypertension (Girardin et al. 1980). These infiltrating immune cells release pro‐inflammatory cytokines, including IL‐17A, IFN‐γ, IL‐6, and TNF‐α, ROS, and other mediators that promote oxidative stress, endothelial dysfunction, and local activation of RAAS, ultimately increasing renal vascular resistance, augmenting tubular sodium reabsorption, and further impairing pressure–natriuresis (Girardin et al. 1980). Notably, such immune cell accumulation and inflammatory amplification are markedly attenuated in salt‐resistant strains, supporting a genetically determined susceptibility to sodium‐induced immune activation and hypertensive disease (Girardin et al. 1980).

Building on these findings, a human translational study investigated whether RAAS signaling within myeloid immune cells triggers salt‐induced immune activation, using both human monocytes exposed to high sodium ex vivo and circulating myeloid cells from hypertensive patients undergoing salt loading and depletion (Ertuglu et al. 2023). Although high sodium altered several RAAS‐related genes in isolated monocytes, including reduced expression of renin and renin‐binding protein and increased expression of natriuretic peptide A, these changes were not reproduced in vivo (Ertuglu et al. 2023). Dietary salt manipulation did not significantly alter RAAS‐related gene expression in circulating myeloid cells in either salt‐sensitive or salt‐resistant individuals (Ertuglu et al. 2023). This discrepancy may reflect differences between sustained, controlled sodium exposure in isolated cells and the more tightly regulated extracellular sodium environment in vivo. Additionally, these findings may suggest that RAAS‐related gene expression is unlikely to mediate salt‐induced myeloid immune activation in salt‐sensitive hypertension. Further studies are therefore needed to define the mechanisms regulating salt‐induced antigen‐presenting cell activation and their contribution to downstream inflammation and end‐organ damage.

Autonomic modulation is central to blood pressure homeostasis during sodium loading, yet, in salt‐sensitive individuals, compensatory mechanisms falter. Whereas normal autonomic regulation triggers enhanced baroreflex sensitivity and suppressed sympathetic outflow in response to sodium‐mediated volume overload, salt‐sensitive individuals display impaired baroreflex and persistent sympathetic activity. This failure not only perpetuates hypertension but also increases cardiovascular risk through sustained neurohumoral activation and vascular injury. Furthermore, excessive sodium intake induces endothelial dysfunction and structural remodeling of both small and large arteries, reducing NO bioavailability, elevating arterial stiffness, and amplifying systemic vascular resistance (Nista et al. 2020). These multifactorial maladaptation's, stretching from renal to vascular and autonomic domains, underpin the sustained blood pressure elevation observed in salt‐sensitive hypertension.

## 7 Alterations in Gut Microbiota Composition

Recent evidence has firmly established that alteration in gut microbiota composition, termed dysbiosis, is implicated in the pathogenesis of hypertension (O'Donnell et al. 2023). Indeed, it is becoming increasingly apparent that gut microbiota contributes to blood pressure regulation through an intricate network of metabolic, immune, and neurohumoral interactions (Figure 8). Commensal bacteria ferment indigestible dietary fibers into short‐chain fatty acids (SCFAs), including acetate, propionate, and butyrate, that exert vasoregulatory and immunomodulatory actions by activating G‐protein‐coupled receptors such as GPR41, GPR43, and Olfr78, directly affecting vascular tone, renal renin secretion and local or systemic inflammation (Marques et al. 2018). Furthermore, these bacterial metabolites modulate gene expression pathways by inhibiting histone deacetylases, thereby shaping inflammatory signaling and the circadian regulation of blood pressure (Marques et al. 2018). Indeed, high‐fiber dietary patterns, which enrich SCFA‐producing bacteria, are consistently associated with reduced systemic inflammation, improved endothelial function, and attenuated RAAS activity, collectively maintaining blood pressure within normal range (Estruch et al. 2009; S. P. Whelton et al. 2005).

**Figure 8:**
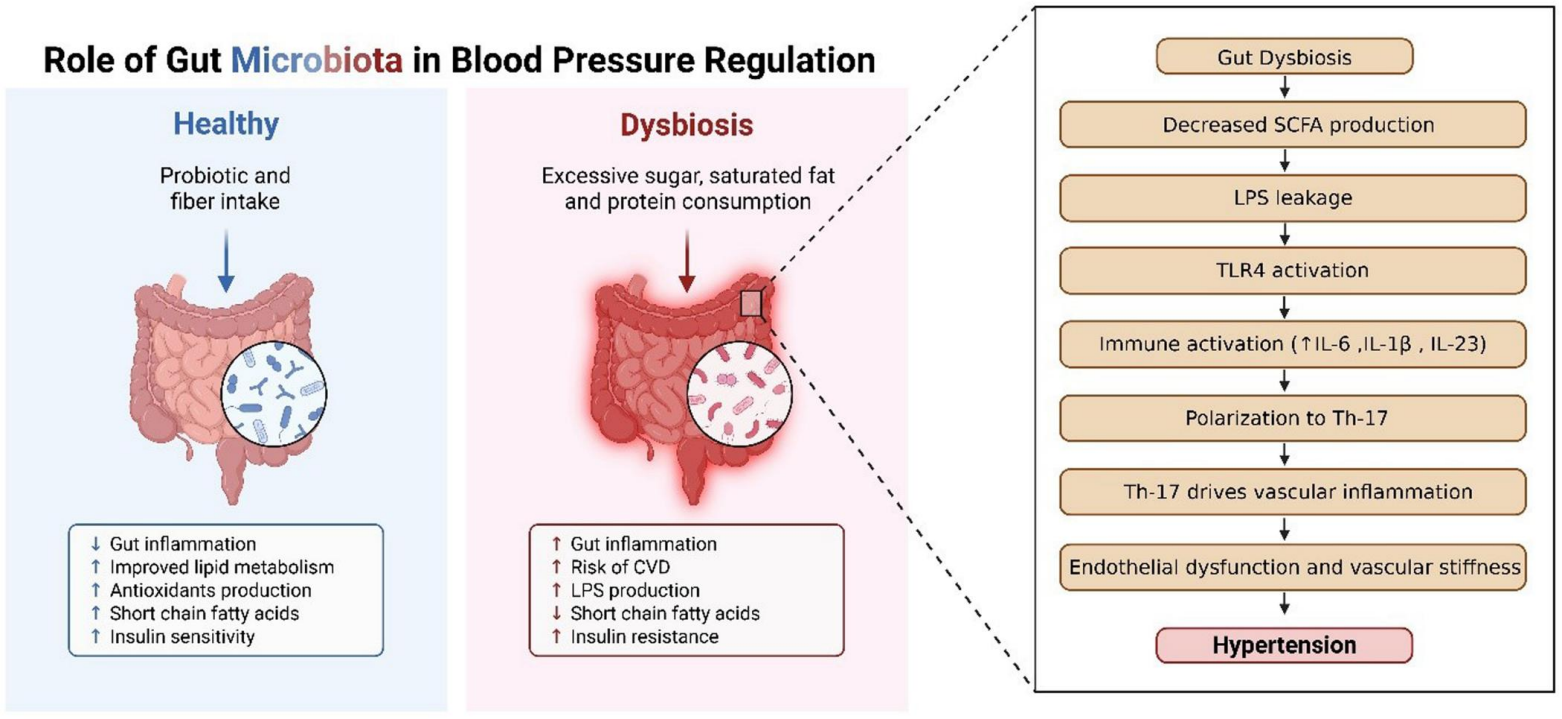
Role of gut microbiota in blood pressure regulation and hypertension. A balanced gut microbiota, supported by fiber‐rich diets, promotes anti‐inflammatory effects, improved lipid metabolism, increased antioxidant production, and enhanced insulin sensitivity, all of which contribute to vascular health and blood pressure regulation. In contrast, dysbiosis, often resulting from excessive intake of sugar, saturated fats, and animal protein, leads to reduced SCFA production and increased intestinal permeability. This permits the translocation of lipopolysaccharides (LPS) into the circulation, where they activate toll‐like receptor 4 (TLR4) on immune cells. TLR4 activation triggers the release of pro‐inflammatory cytokines (IL‐6, IL‐1β, IL‐23) and promotes polarization of T‐helper cells toward the Th‐17 phenotype. Th‐17 cells drive vascular inflammation, resulting in endothelial dysfunction and increased vascular stiffness. These changes collectively contribute to the development of hypertension. Created with BioRender.com.

The state of microbiota dysbiosis, typified by a loss of beneficial microbes, proliferation of potentially pathogenic species, and a decline in overall microbial diversity, has profound consequences for host metabolic and cardiovascular health (DeGruttola et al. 2016). Diet is one of the primary determinants of gut microbial balance, with excessive consumption of fat, sugar, or processed foods, and inadequate fiber intake, resulting in the expansion of harmful bacteria and a reduction in the production of protective metabolites like SCFAs (Marques et al. 2018). This imbalance fosters systemic inflammation and immune dysfunction, culminating in an increased risk for a range of chronic diseases, including hypertension, obesity and atherosclerosis (DeGruttola et al. 2016). Hence, dietary modulation represents a tangible mechanism to restore microbial composition and forestall adverse cardiovascular outcomes.

Experimental studies using germ‐free (GF) or gnotobiotic mice have conclusively demonstrated the causal relationship between gut microbial signals and the regulation of blood pressure. These mice exhibit lower baseline blood pressure and blunted hypertensive responses to stimuli such as angiotensin II, compared to conventionally colonized counterparts (Karbach et al. 2016; Moghadamrad et al. 2015). Reintroducing gut microbiota into GF mice not only normalized blood pressure but also corrected the underlying vascular dysfunction observed in the absence of microbes. This microbial restoration was linked to enhanced actin polymerization in VSMCs, mediated by the phosphorylation and subsequent inactivation of cofilin, an actin‐depolymerizing protein. Stabilization of actin filaments improves vascular contractility independent of systemic catecholamine levels, underscoring a direct microbiota–vascular signaling pathway rather than one solely dependent on neurohumoral control. These findings highlight a novel, microbiota‐dependent pathway for blood pressure regulation that operates at the level of the vascular cytoskeleton rather than hormonal control.

To confirm causality, fecal microbiota transfer (FMT) was conducted from hypertensive donors to normotensive controls, revealing a marked elevation in their blood pressure (Adnan et al. 2017; de la Visitación et al. 2021; Li et al. 2017). Conversely, performing FMT from normotensive donors to hypertensive recipients led to a significant reduction in their blood pressure (Toral et al. 2019). Notably, these observations have been attributed to several underlying mechanisms, underscoring the complex interplay through which microbiota dysbiosis contributes to the pathogenesis of hypertension. A key pathway involves inflammation driven by LPS, a pro‐inflammatory endotoxin abundantly expressed within dysbiotic microbiota (Figure 8) (Li et al. 2017). Mechanistically, microbiota dysbiosis compromises the integrity of intestinal epithelial barrier, enabling leakage of LPS into the systemic circulation (O'Donnell et al. 2023). In turn, LPS activates TLR4 signaling, a central pathway of innate immunity, triggering low‐grade systemic inflammation, endothelial dysfunction, and vascular stiffness, which collectively contribute to the development of hypertension (Li et al. 2017; O'Donnell et al. 2023). Beyond its peripheral effects, LPS also targets the PVN of the hypothalamus, a central hub for autonomic blood pressure regulation (Toral et al. 2019). Within the PVN, LPS stimulates ROS production by NADPH oxidase and upregulates cytokines such as TNF‐α, IL‐6, and IL‐17 A [76]. Collectively, these inflammatory and oxidative signals in the PVN increase sympathetic outflow, as evidenced by elevated plasma norepinephrine levels (Toral et al. 2019). This neuroimmune interplay contributes to sustained vasoconstriction and increased cardiac output, thereby elevating blood pressure.

The Firmicutes‐to‐Bacteroidetes (F:B) ratio, a frequently cited hallmark of microbiota dysbiosis, serves as an indicator of shifts in gut microbial ecology (Adnan et al. 2017). An elevated F:B ratio, seen in both hypertensive animals models and patients, reflects an enrichment in lactate‐producing *Lactobacillus*, a Firmicute positively associated with hypertension, and a depletion of butyrate‐ and acetate‐producing taxa such as *Clostridiaceae*, *Holdemania*, and *Coprobacillus*, which are negatively associated with hypertension (Adnan et al. 2017). The resultant decline in beneficial SCFAs production, including butyrate, propionate, and acetate, diminishes the activation of GPR41 and GPR43 on vascular and renal tissues, reduces Histone deacetylase inhibition, and weakens anti‐inflammatory signals (Adnan et al. 2017; Jama et al. 2019; Li et al. 2017). While not a clinical biomarker, the F:B ratio underscores the reproducible link between gut microbial composition, hypertension, and vascular health.

The gut microbiota's interface with immune function represents an additional regulatory axis for blood pressure homeostasis. Studies in GF animals show that lack of microbiota results in reduced T‐helper 1 (Th1) immune responses, lower adhesion and infiltration of inflammatory leukocytes, and protection against vascular oxidative stress and renal tissue damage under hypertensive stimuli (Karbach et al. 2016). Dysbiosis, by promoting the expansion of antigen‐presenting DCs and macrophages, fosters increased production of cytokines such as IL‐6, IL‐1β, and IL‐23. These cytokines polarize naive T cells toward a Th‐17 phenotype, resulting in elevated IL‐17A secretion and further ROS generation (de la Visitación et al. 2021) (Figure 8). Through this cascade, vascular NO bioavailability is diminished, vasodilation impaired, and arterial stiffness increased, together perpetuating blood pressure elevation.

## 8 Disruption of Circadian Rhythm

The body's circadian rhythm is a fundamental biological framework that governs a wide array of physiological and behavioral functions, including the sleep–wake cycle, hormone secretion, thermoregulation, metabolic functions, and critically, blood pressure regulation. This rhythm is orchestrated by the suprachiasmatic nucleus (SCN) of the hypothalamus, often described as the master circadian clock (Figure 9) (Hastings et al. 2018). Situated bilaterally above the optic chiasm, the SCN comprises two variegated regions, the core and the shell (Hastings et al. 2018). These regions collaboratively process photic signals via the retinohypothalamic tract originating from intrinsically photosensitive retinal ganglion cells. These photic inputs are integrated primarily in the shell, which orchestrates peripheral circadian clocks by transmitting temporal cues through neurotransmitters, notably gamma‐aminobutyric acid (GABA), along with paracrine mediators (Reddy et al. 2025). This hierarchical arrangement ensures synchronization among organ‐specific clocks, optimizing physiological function across the diurnal cycle. Although secondary cues like body temperature fluctuations and feeding patterns can modulate peripheral clocks, their influence is subordinate to SCN‐mediated central entrainment, underscoring the SCN's paramount role in temporal homeostasis (Mohawk et al. 2012).

**Figure 9:**
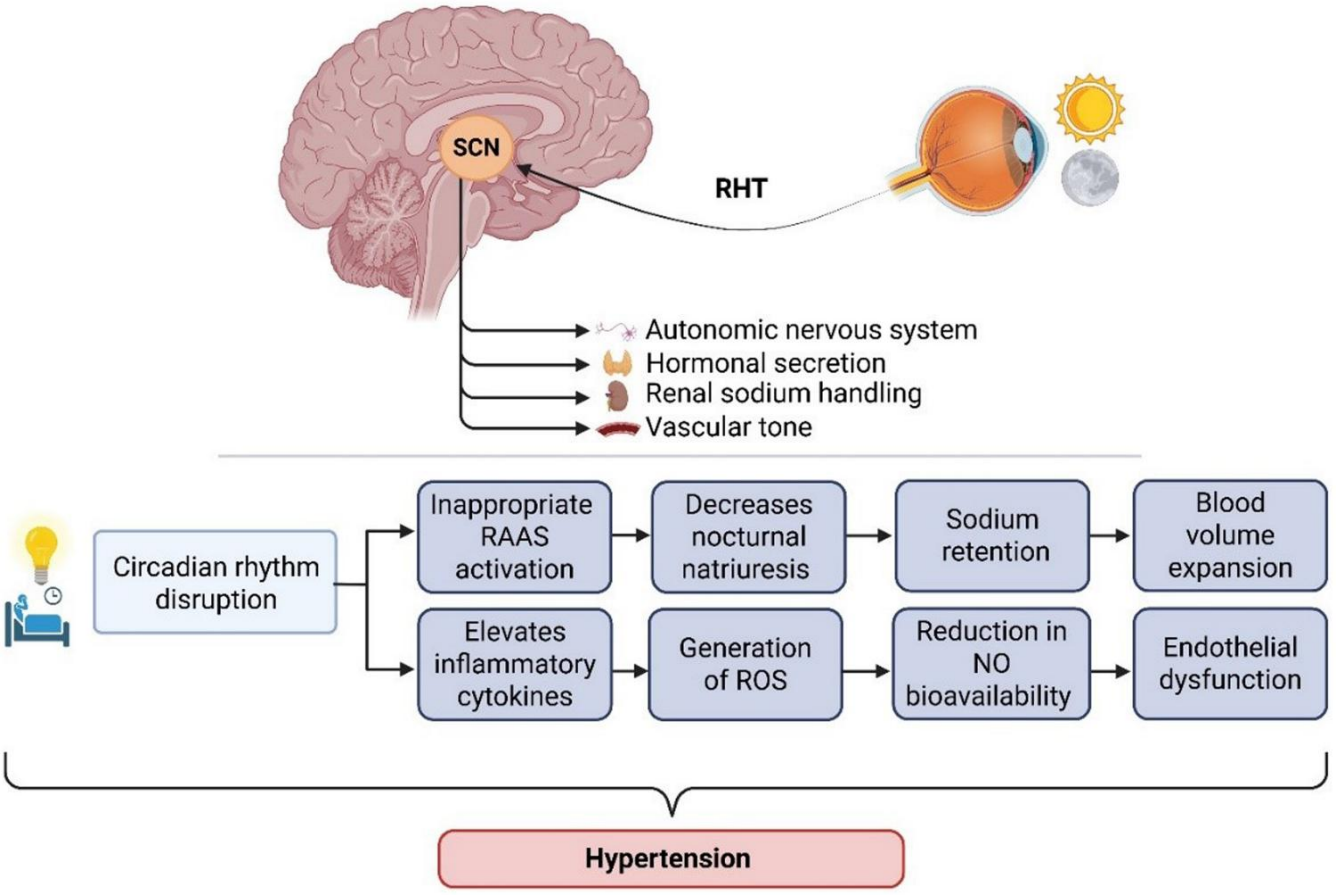
Circadian rhythm disruption in hypertension. Light cues received by the retina are transmitted via the retinohypothalamic tract (RHT) to the suprachiasmatic nucleus (SCN), the brain's central circadian pacemaker. The SCN regulates key physiological processes, autonomic nervous system activity, hormonal secretion, renal sodium handling, and vascular tone, in a time‐dependent manner. Disruption of circadian rhythms, due to altered sleep–wake cycles or irregular light exposure, impairs these regulatory pathways and contributes to inappropriate activation of the renin‐angiotensin‐aldosterone system (RAAS), reduced nocturnal natriuresis, sodium retention, and blood volume expansion. Simultaneously, circadian misalignment elevates inflammatory cytokine production and promotes reactive oxygen species (ROS) generation, leading to decreased nitric oxide (NO) bioavailability and endothelial dysfunction. Together, these mechanisms converge to increase vascular resistance and promote the development of hypertension. Created with BioRender.com.

Blood pressure manifests a robust circadian rhythm orchestrated by the SCN through synchronization of peripheral clocks resident in key blood pressure–regulating organs, including the heart, vasculature, kidneys, and adrenal glands (Hastings et al. 2003). This rhythm is characterized by a daytime elevation in blood pressure, a 10%–20% nocturnal decline during sleep, and a morning surge aligned with awakening. At the molecular level, circadian regulation relies on transcription–translation feedback loops (TTFLs) involving core clock genes such as brain and muscle ARNT‐like 1 (BMAL1), circadian locomotor output cycles protein kaput (CLOCK), period (PER), and cryptochrome (CRY), which generate self‐sustaining ~24‐h oscillations in gene expression. BMAL1 and CLOCK act as the principal activators in the circadian loop, driving the transcription of PER and CRY during the “day” phase. This activation promotes wakefulness and daytime physiological functions, including increased sympathetic activity, higher metabolic rate, and elevated blood pressure necessary for active periods (Hastings et al. 2018). As PER and CRY proteins accumulate, they inhibit BMAL1–CLOCK activity, initiating the “night” phase. This period is characterized by reduced neuronal excitability, lower metabolic demand, and vasodilation, which collectively contribute to the nocturnal drop in blood pressure, facilitating sleep and tissue recovery (Hastings et al. 2018). This oscillatory mechanism also governs clock‐controlled genes (CCGs) that modulate physiological processes including vascular tone, renal sodium handling, and neurohumoral activity (Faraci and Scheer 2024). Notably, temporal information relayed from SCN via neuronal and hormonal pathways mainly influences sympathetic nervous system output and the RAAS (Douma and Gumz 2018). Collectively, these mechanisms ensure alignment between cardiovascular function and behavioral cycles, optimizing perfusion during daytime activity and conserving energy during nighttime rest.

Disruption of circadian blood pressure rhythmicity, manifested as blunted nocturnal dipping, reverse dipping, or exaggerated morning surges, is strongly associated with hypertension and cardiovascular morbidity (Hastings et al. 2003). Factors such as shift work, irregular sleep–wake patterns, or chronic exposure to artificial light provoke circadian misalignment, leading to desynchronization between central SCN and peripheral clocks, and impaired vascular, renal, and neurohormonal regulation (Faraci and Scheer 2024). Experimental desynchronization paradigms demonstrate a modest but clinically meaningful elevation in mean arterial pressure (+ 3 mmHg), with epidemiologic correlations to increased cardiovascular risk paralleling those seen in dietary intervention studies (Scheer et al. 2009). Notably, evidence implicates impaired renal sodium excretion during the usual nocturnal period, attributable to core clock gene deficits, in promoting volume overload and salt sensitivity. Human studies corroborate these findings, with primary hyperaldosteronism patients exhibiting diminished nocturnal pressure declines and associated adverse cardiac remodeling. These observations delineate the central pathological role of circadian‐driven renal sodium handling in hypertension (Costello and Gumz 2021) (Figure 9). This experimental evidence aligns with human studies showing that nondipping patterns correlate with activation of RAAS (Q. Wu et al. 2021). These findings underscore that the key pathology lies in the timing of sodium handling rather than total excretion, with impaired nocturnal natriuresis serving as a central driver of hypertension.

Circadian misalignment provokes a systemic proinflammatory state, evidenced by increased circulating cytokines and acute phase proteins such as IL‐6, TNF‐α, CRP, and resistin. These mediators impair endothelial function, enhance vasoconstriction, and contribute to autonomic imbalances such as decreased cardiac vagal tone and increased catecholamine excretion, collectively culminating in elevated nocturnal blood pressure (Figure 9). Such mechanistic insights were derived from controlled human studies simulating shift work schedules, where circadian inversion closely paralleled surges in inflammatory and sympathetic biomarkers, delineating a multifaceted pathophysiological cascade linking circadian disruption with hypertension (Morris et al. 2016). Intriguingly, circadian perturbations may yield divergent hemodynamic outcomes. Loss‐of‐function animal models targeting clock genes BMAL1 or PER reveal phenotypes ranging from hypertension to hypotension, depending on tissue‐specific expression and downstream signaling interactions (Curtis et al. 2007). For example, smooth muscle‐specific BMAL1 deletion attenuates vasoconstriction and blunts normal diurnal blood pressure rises, whereas adipose tissue–targeted deletions disrupt angiotensinogen expression and produce exaggerated nocturnal pressure dips (Faraci and Scheer 2024). These findings highlight the complexity of clock gene regulation and necessitate further investigation to delineate molecular determinants guiding phenotypic divergence.

## 9 Insulin Resistance

Insulin plays a central role in maintaining plasma glucose homeostasis. Beyond its metabolic actions, insulin contributes to vascular tone, by influencing the production of key vasoactive metabolites (Fu et al. 2021). This dual functionality is mediated primarily by insulin receptors (IR) expressed on vascular endothelial cells and VSMCs. These receptors enable insulin to orchestrate complex signaling pathways that regulate vascular reactivity and systemic blood pressure. Thus, insulin plays an essential role in aligning metabolic demand with vascular function, establishing a crucial link between metabolic health and cardiovascular regulation (Fu et al. 2021).

Binding of insulin to IR initiates phosphorylation of insulin receptor substrates (IRS‐1/2), triggering the phosphoinositide 3‐kinase (PI3K)/Akt pathway, which in turn phosphorylates and activates eNOS. This cascade enhances NO production, promoting VSMC relaxation and improving blood flow to metabolically active tissues (Fu et al. 2021). In parallel, insulin also influences vascular tone by activating myosin light chain phosphatase (MLCP) in VSMCs, facilitating muscle relaxation (Cooper et al. 2007). Simultaneously, insulin engages the MAPK pathway, which governs vascular growth and remodeling, and can stimulate vasoconstrictive mediators such as endothelin‐1 (Cooper et al. 2007). Under physiological conditions, the vasodilatory actions of the PI3K/Akt axis predominate, while MAPK‐driven proliferation maintains vascular integrity and adaptive remodeling (Cooper et al. 2007). This balanced interplay ensures fine‐tuned regulation of vascular tone and structure to meet metabolic demands while preserving homeostasis. Insulin resistance disrupts this delicate vascular balance by selectively impairing the PI3K/Akt–eNOS pathway while sparing or even exaggerating the MAPK signaling cascade (Brosolo et al. 2022). This “selective resistance” reduces NO bioavailability, thereby blunting vasodilation and predisposing to endothelial dysfunction (Kim et al. 2006). Simultaneously, intact MAPK signaling augments vasoconstrictor, pro‐inflammatory, and proliferative responses in VSMCs, further amplifying vascular tone and remodeling (Jiang et al. 1999). This selective molecular dysregulation effectively links metabolic insulin resistance with heightened vascular reactivity and hypertensive pathophysiology.

Beyond vascular effects, insulin directly influences renal sodium handling by virtue of the widespread distribution of IRs along nephrons (Tedde et al. 1988). Indeed, insulin enhances sodium reabsorption at multiple tubular segments, including the proximal tubule, the thick ascending limb of Henle's loop, and distal tubule. This effect is mediated through activation of transporters such as the Na^+^/H^+^ exchanger (NHE3) and Na^+^/K^+^‐ATPase in the proximal tubule, along with the NKCC2 and ENaC in the thick ascending limb and distal tubule, respectively (Tedde et al. 1988). Furthermore, dietary sodium manipulations have been shown to modulate renal IR density, with high‐salt intake reducing receptor binding across several tubular segments, underscoring insulin's regulatory role in renal sodium reabsorption (Sechi et al. 1991). Genetic knockout models targeting renal IRs corroborate these mechanisms, showing impaired natriuresis, increased salt sensitivity, and altered blood pressure regulation (Brosolo et al. 2022). Collectively, these data highlight insulin's role in renal sodium balance and its contribution to blood pressure homeostasis.

Hyperinsulinemic–euglycemic clamp studies in humans, the gold standard for assessing insulin sensitivity, were used to evaluate the role of insulin and the effect of hyperinsulinemia on sodium balance. Such studies involve maintaining high plasma insulin levels via infusion while “clamping” blood glucose at normal levels through variable glucose infusion, with the glucose infusion rate reflecting tissue responsiveness to insulin (DeFronzo et al. 1975). By isolating the effects of insulin from confounding glycemic fluctuations, it was shown that hyperinsulinemia induces significant sodium retention, particularly at distal nephron segments (DeFronzo et al. 1975). This was further supported by evidence from normotensive individuals with family history of hypertension, in whom hyperinsulinemia induced using the same technique reduced urinary sodium excretion by approximately 50% (Herlitz et al. 1996). Notably, the antinatriuretic effect of insulin was also evident in individuals with type 2 diabetes, with reductions in fractional sodium excretion increasing with the dose of insulin infused (Gans et al. 1992). These findings not only confirm insulin's role in modulating renal sodium reabsorption but also underscore that hyperinsulinemia, a hallmark of insulin resistance, promotes sodium retention, thereby contributing to elevations in blood pressure.

Compensatory hyperinsulinemia also contributes to the development of hypertension through other mechanisms. Particularly, hyperinsulinemia enhances sympathetic nervous system activity, through central and peripheral mechanisms, further elevating peripheral resistance and contributing to renal vasoconstriction (da Silva et al. 2020). Centrally, insulin crosses the blood–brain barrier and acts on hypothalamic nuclei, specifically the arcuate and paraventricular nuclei, stimulating sympathetic outflow (Stocker and Gordon 2015). This results in increased norepinephrine release to peripheral vasculature and the kidneys, leading to arteriolar vasoconstriction and elevated peripheral resistance (Stocker and Gordon 2015). Peripherally, insulin augments renal sympathetic nerve activity, which heightens renin release and promotes sodium reabsorption, thereby increasing intravascular volume (Brosolo et al. 2022; da Silva et al. 2020). Together, these effects create a sustained rise in blood pressure and contribute to the vascular remodeling observed in insulin‐resistant states.

Insulin resistance further exacerbates vascular injury via dysregulated adipokine secretion and elevated free fatty acid (FFA) flux. Normally, insulin suppresses adipose tissue lipolysis; however, in insulin resistance, this inhibition is lost, accelerating FFA release (Sakr et al. 2023). The resulting lipid spillover into the liver and muscle exacerbates systemic insulin resistance and promotes pro‐inflammatory signaling in adipocytes and macrophages. This shifts the adipokine profiles toward increased resistin, leptin, TNF‐α, and IL‐6, while levels of the anti‐inflammatory adiponectin decline (Pulakat et al. 2012). Resistin is particularly implicated as it activates NF‐κB and MAPK pathways via TLR4 signaling. This, in turn, upregulates adhesion molecules, particularly VCAM‐1 and ICAM‐1, along with cytokines such as TNF‐α and IL‐6, thereby fostering endothelial dysfunction and vascular inflammation (Jamaluddin et al. 2012). Concurrently, resistin reduces eNOS expression and NO bioavailability, favoring vasoconstriction and arterial stiffness, while also enhancing smooth muscle proliferation and oxidative stress (Fang et al. 2013; Jamaluddin et al. 2012). Leptin also contributes by augmenting sympathetic outflow and RAAS activation, promoting further vascular remodeling and sodium retention (Sabbatini et al. 2015). These inflammatory and hemodynamic perturbations collectively precipitate vascular stiffening, culminating in hypertension.

Persistent hyperglycemia, a hallmark of insulin resistance and type 2 diabetes, accelerates vascular dysfunction through advanced glycation end‐product (AGE) formation (Ohishi 2018). These AGEs, in turn, progressively accumulate within the vascular extracellular matrix, where they induce structural cross‐linking of matrix proteins, thereby reducing arterial compliance and impairing vascular elasticity (Petrie et al. 2018). This process is compounded by ROS generation, establishing a self‐amplifying cycle of oxidative stress that further accelerates AGE formation (Petrie et al. 2018). Engagement of AGEs with their receptor (RAGE) activates multiple redox‐sensitive signaling cascades, including NF‐κB, MAPK, transforming growth factor‐β (TGF‐β), and NADPH oxidases (Petrie et al. 2018). These cascades upregulate vascular adhesion molecules, including VCAM‐1 and E‐selectin, vascular endothelial growth factors, and pro‐inflammatory cytokines, including IL‐1β, IL‐6, and TNF‐α. Together, these factors foster endothelial dysfunction, inflammation, and vascular fibrosis. Consequently, These changes collectively elevate peripheral vascular resistance and reduce vasodilatory capacity, establishing a foundation for sustained hypertension (Petrie et al. 2018).

Clinical data reinforces the intertwined nature of insulin resistance and hypertension, with up 50% of patients with essential hypertension displaying hyperinsulinemia or impaired glucose tolerance, and a majority of patients with type 2 diabetes developing hypertension (Zhou et al. 2014). This overlap reflects shared upstream drivers, including visceral adiposity, Western dietary patterns, and sedentary behavior, thereby underscoring the bidirectional reinforcement between metabolic and hemodynamic derangements (Brosolo et al. 2022). Therefore, insulin resistance is not merely an epiphenomenon of obesity but a central mechanistic contributor to elevated blood pressure, operating through vascular, renal, neurohormonal, and inflammatory pathways that collectively cause and sustain hypertension.

## 10 Conclusion and Perspectives

The multifactorial drivers of hypertension underscore the limitations of reductionist, single‐target therapeutic strategies and support a transition toward integrated frameworks capable of simultaneously addressing central and peripheral regulatory mechanisms. Indeed, the RAAS, CNS dysfunction, and inflammatory‐oxidative stress pathways represent core pathological axes whose reciprocal interactions sustain blood pressure elevation. These pathways converge with modifiable factors like dietary patterns, circadian disruption, gut microbiota dysbiosis, and insulin resistance, which amplify disease progression. Owing to this complex interdependence, therapeutic strategies must transcend symptomatic blood pressure reduction to address upstream drivers, enhancing treatment durability and cardiovascular risk mitigation.

Future investigations should therefore prioritize the elucidation of the hierarchical interplay and temporal dynamics among these complex mechanisms to develop targeted, personalized interventions. By virtue of advances in multi‐omics technologies, systems biology, computational modeling, we shall be increasingly able to delineate the molecular networks that integrate neurohumoral, metabolic and immune signaling in hypertension. Longitudinal cohort combined with high‐resolution molecular profiling may enable the identification of predictive biomarkers and novel therapeutic targets within these interconnected pathways. Clinically, the integration of lifestyle modifications with pharmacological agents that influence circadian regulation, immune activation, and gut microbiota may offer a more comprehensive strategy for restoring vascular homeostasis. Within this broader therapeutic arena, natural‐product pharmacology provides valuable mechanistic insights; experimental studies have shown that extracts of and bioactives derived from medicinal plants exert various cardio‐vasculoprotective effects, including endothelium‐dependent vasorelaxation (Jomova et al. 2025; Wan et al. 2025). This highlights the potential of naturally occurring molecular as modulators of endothelial function and vascular health.

Within this therapeutic landscape, immune‐targeted therapies represent a promising avenue for mitigating hypertension. Although clinical evidence remains limited, immunomodulatory agents, including mycophenolate mofetil and TNF‐α inhibitors, have been incidentally associated with blood pressure reductions, providing preliminary support for the therapeutic relevance of immune modulation in hypertension. Nevertheless, the interpretation of these findings remains constrained by the fact that available studies were predominantly conducted in patients receiving immunomodulatory therapy for underlying autoimmune or inflammatory diseases rather than hypertension itself, with many participants being normotensive at baseline. Moreover, the potential benefits of systemic immunosuppression must be carefully balanced against clinically relevant risks, particularly susceptibility to serious infections, which may outweigh modest reductions in blood pressure given the established efficacy and favorable safety profiles of conventional antihypertensive therapies. Future investigations should therefore move beyond broad immunosuppression toward the selective modulation of immune pathways directly implicated in hypertensive pathophysiology (Tluli et al. 2026). Advances in immune profiling and molecular phenotyping may facilitate the identification of inflammatory hypertension endotypes most likely to benefit from such interventions, while adequately powered randomized controlled trials specifically enrolling hypertensive populations will be essential to establish their efficacy, safety, and effects on hypertension‐mediated organ damage (Abdelhamid et al. 2026). The *raison d'être* of chronotherapy lies in synchronizing drug administration with endogenous biological cycles to optimize efficacy and minimize adverse effects. Microbiota‐targeted interventions, including prebiotics, probiotics, and fecal microbiota transplantation, warrant rigorous evaluation for their capacity to modulate immune and neurohumoral dysfunction in hypertension. Collectively, these holistic approaches hold potential to revolutionize hypertension management, reduce global cardiovascular morbidity, and tailor care to individual pathophysiological profiles.

## Author Contributions

**Dima Nasrallah:** writing – original draft. **Alaa Abdelhamid:** writing – review and editing. **Omar Tluli:** writing – review and editing. **Yaman Al‐Haneedi:** writing – review and editing. **Mera Abu Hussien:** writing – review and editing. **Ali H. Eid:** writing – original draft, writing – review and editing, supervision, project administration.

## Ethics Statement

The authors have nothing to report.

## Consent

The authors have nothing to report.

## Conflicts of Interest

The authors declare no conflicts of interest.

## Data Availability

The authors have nothing to report.
